# The E-Textile for Biomedical Applications: A Systematic Review of Literature

**DOI:** 10.3390/diagnostics11122263

**Published:** 2021-12-03

**Authors:** Giuseppe Cesarelli, Leandro Donisi, Armando Coccia, Federica Amitrano, Giovanni D’Addio, Carlo Ricciardi

**Affiliations:** 1 Department of Chemical, Materials and Production Engineering, University of Naples “Federico II”, 80125 Naples, Italy; giuseppe.cesarelli@unina.it; 2 Bioengineering Unit, Institute of Care and Scientific Research Maugeri, 82037 Pavia, Italy; leandro.donisi@unina.it (L.D.); armando.coccia@unina.it (A.C.); gianni.daddio@icsmaugeri.it (G.D.); carloricciardi.93@gmail.com (C.R.); 3 Department of Advanced Biomedical Sciences, University of Naples “Federico II”, 80131 Naples, Italy; 4 Department of Electrical Engineering and Information Technologies, University of Naples “Federico II”, 80125 Naples, Italy

**Keywords:** e-textile, health monitoring, diagnosis, wearable, biomedical engineering, sEMG, ECG, smart garments, motion analysis, IMUs

## Abstract

The use of e-textile technologies spread out in the scientific research with several applications in both medical and nonmedical world. In particular, wearable technologies and miniature electronics devices were implemented and tested for medical research purposes. In this paper, a systematic review regarding the use of e-textile for clinical applications was conducted: the Scopus and Pubmed databases were investigate by considering research studies from 2010 to 2020. Overall, 262 papers were found, and 71 of them were included in the systematic review. Of the included studies, 63.4% focused on information and communication technology studies, while the other 36.6% focused on industrial bioengineering applications. Overall, 56.3% of the research was published as an article, while the remainder were conference papers. Papers included in the review were grouped by main aim into cardiological, muscular, physical medicine and orthopaedic, respiratory, and miscellaneous applications. The systematic review showed that there are several types of applications regarding e-textile in medicine and several devices were implemented as well; nevertheless, there is still a lack of validation studies on larger cohorts of subjects since the majority of the research only focuses on developing and testing the new device without considering a further extended validation.

## 1. Introduction

Wearable technology includes devices that consumers can comfortably wear and use for extended periods of time in an unobtrusive way, like clothing or accessories, with the aim of collecting the data of users’ personal health or, more generally, of interfacing with the user. The predominant category of wearable devices in the current market consists of, by far, smart accessories including smartwatches, wristbands, smart glasses, and various clothing clip-ons [1]. These accessories typically rely on existing miniature sensors and electronics enclosed in compact items that can be worn. However, their structure makes them rigid and nonflexible and, consequently, not ideal for the development of more advanced wearable systems that need larger contact and interface with user’s body. The integration of micro- and nano-electronics in textile substrates can be relevant for the development of more ergonomic smart materials, which are broadly known as electronic textiles (e-textiles). Through e-textile technology, a wide spectrum of functions, found in rigid and nonflexible electronic products nowadays, can be potentially developed on a textile substrate [2].

This attractive opportunity aroused a great deal of interest in wearable e-textile devices. The market of wearable technologies has a compound annual growth rate of 15.5%, which is expected to further continue thanks to the rapid improvements in technology and miniature devices, as well as mobile computing. In a recent market forecast the industry of wearable devices is estimated to grow to more than $US155 billion by 2027 [3], with the involvement of many big companies which are multiplying their research efforts to shift from the wearable electronic hardware to the more comfortable e-textiles.

The growing demand from consumers is encouraging manufacturers to produce and sell billions of wearable electronic products, covering various sectors of the market, including health and wellness, military and defense, space exploration, fashion, and entertainment. Healthcare is identified as one of the most promising market, piloted by the increasing desire of consumers to continually monitor their own health and by the interest of healthcare professionals to have more health data at their disposal to better examine a larger cohort of patients.

The comfort, ease-of-use, and ubiquity offered by smart biomedical clothes potentially represent key factors for the continuous long-term clinical monitoring. The integration of these innovative devices in Internet of Things (IoT) networks, exploiting simple but efficient wireless solutions, makes it possible to establish smart systems for remote health monitoring, allowing patients to continue to stay at home rather than in expensive healthcare facilities. One of the main purposes of a wearable health monitoring system is to ensure continuous, noninvasive, and seamless surveillance of health and physical well-being, enabling people to lead independent and active lives in their familiar home environment [4]. This is a great advantage especially for patients with chronic diseases and/or with mobility difficulties. The use of wearable monitoring systems underlines two other benefits for users: firstly, it reduces the influence and stress that the clinical environment exerts on patient’s performance [5]; secondly, the great amount of data gathered with this system can be processed using Artificial Intelligence (AI) algorithms to detect a possible worsening of a patient’s clinical situation [6].

From the public health system perspectives, the development of smart wearable biomedical systems has the potential to offer advanced services to patients, combining the frequently worn material with the most technologically advanced, sensing, processing and communicating capabilities [7], and, at the same time, to support health cost reduction by facilitating early hospital discharges. Nevertheless, the great perspectives illustrated clash with the technological limitations that hinder the large-scale production and diffusion of market-ready garments or textiles. These technological challenges justify the remarkable research efforts, which are evidenced by the large number of research prototypes and innovative solutions proposed in the scientific literature.

The main issues to be addressed in the design and fabrication of e-textile systems concern breathability, flexibility, and “washability”, which are fundamental features for comfortable user experience and must be maintained even after integration of the electronic components. Power supply is also a very critical challenge for e-textile devices. Common rechargeable batteries are usually used, though they increase the weight of the devices and are incompatible with the flexibility and washing requirements of textile integration. To overcome these limitations, different functional materials were designed to have different features and approach the goal of self-powered textiles [8]. However, technological innovations should be implemented while ensuring a safe degree of reliability for device performances in comparison with the standard methods commonly used in clinical environment [9].

In addition to technological problems, regulatory issues regarding patient safety, privacy, and data management also represent obstacles to the large commercial diffusion of e-textiles [10,11]. More efforts are needed to develop algorithms to ensure highly secured communication channels in existing low-power, short-range wireless platforms [4].

In summary, it is undeniable that the development of smart textiles requires a multidisciplinary approach in which knowledge and skills in both industrial sciences—e.g., chemistry, design, and fabrication of smart materials—and Information and Communication Technologies (ICT)—e.g., microelectronics and circuit design and fabrication—are fundamentally integrated with a deep understanding of textile arts [2]. Therefore, this review explores the progress in smart e–textiles design and manufacturing, with a focus on biomedical sensors and devices developed for healthcare monitoring. The main aim is to provide a complete overview of the state-of-the-art in this promising area, investigating the various applications and the different approaches and solutions proposed by research groups working on these themes. Indeed, to the best of authors’ knowledge, this is the first systematic review summarizing the research on e-textile for medical applications. Therefore, the choice will be to apply broad criteria for the papers to perform a wider selection and include as many types of papers as possible.

## 2. Materials and Methods

**Figure 1:**
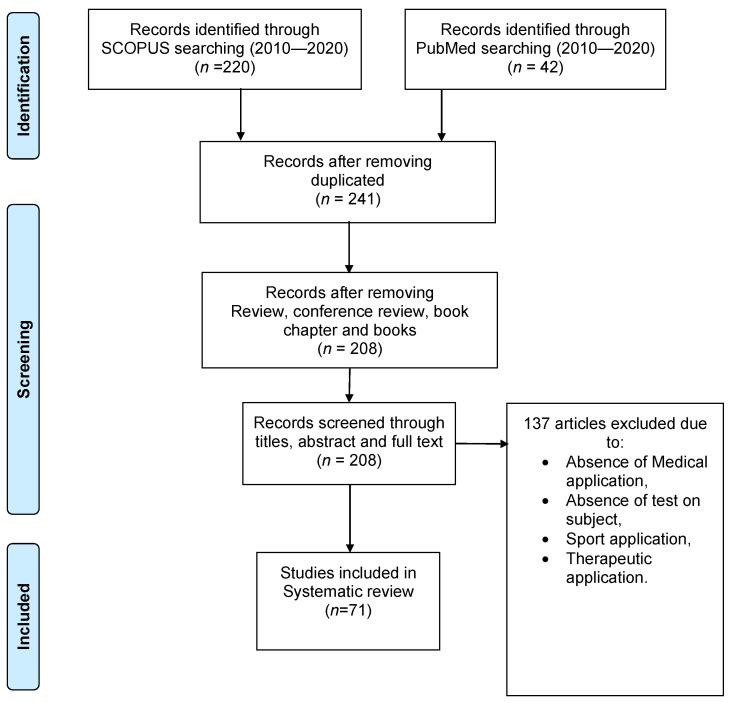
Flow chart for selecting papers from Scopus and Pubmed databases.

The query was conducted on Scopus and Pubmed databases starting from 2010 until 2020 using the words “e-textile”, “textronics”, “textile-electronics” and “monitoring”; 262 articles were found in this time range (23 of them were duplicated). Only English articles were considered, and reviews, conference reviews, book chapters, and books were excluded, thereby reaching 208 papers. Afterwards, all the papers were screened firstly through title and abstract, and then through full text, reaching 71 papers, which were included in this systematic review. Figure 1 shows the whole workflow.

**Figure 2:**
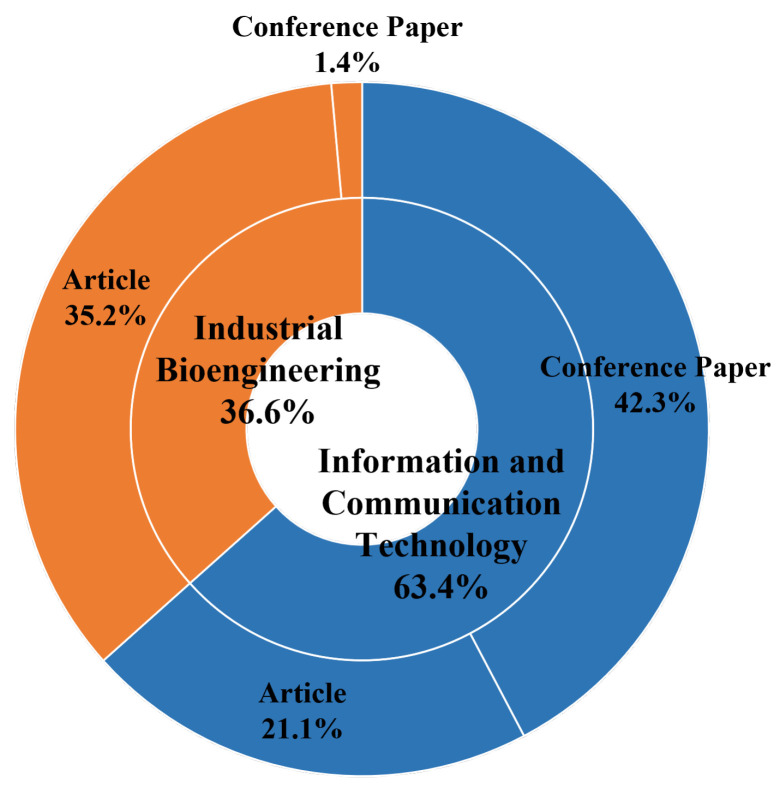
Distribution of papers according to ICT and IB categories, conference papers, and articles.

The articles were categorized into conference papers and articles, Industrial Bioengineering (IB), and ICT domains. Figure 2 depicts the proportions.

## 3. Results

In Table 1 references to the articles included in this review are grouped according to the type of acquired data. The researches are also organized in macro-categories regarding the biomedical field of potential diagnosis. In Table S1 the readers can find more accurate insights regarding each of the articles briefly discussed in the next subsections, where, differently, we propose at the end of each subsection summary tables highlighting, in the first instance, “Aim”, “Dataset” and “Acquired data” for each of the articles investigated.

### 3.1. The Applications in Cardiology

The first line of Table 1 summarized in a concise and schematic form the principal acquired data—in the field of cardiology diagnostics—using e-textile systems.

The electrocardiography signal—called equivalently electrocardiogram (ECG)—was over the years one of the most appropriate tools to diagnose in advance and, consequently, to try to prevent the clinical complications caused by chronic and cardiovascular diseases [83,84]; in recent years, wearable sensors proved to be possible novel alternatives for the ECG acquisition [9], because the e-textiles (used as ECG diagnostic systems) indicated to address—or potentially address—several of the advantages highlighted in Section 1 [83].

The researches in this field are summarized in Table 2. In this 10-year report of papers, the first prototype of ECG e-textile system was presented by Wu et al. [23]. The authors fabricated a cloth electrode into which multiwalled carbon nano-tubes (MWCNTs) were randomly distributed into the fabric, of which one side was connected and fastened with traditional silver/silver chloride (Ag/AgCl) electrodes. The ECG acquisition performed on a single healthy control (HC) demonstrated the novel cloth electrode showed similar performances to the traditional Ag/AgCl electrodes, which might be potentially replaced for the daily and long-term monitoring of the ECG [23]. Similar studies were performed by Acar and Le and the respective coworkers [20,22], which also tested the e-textiles by applying the electrodes on smart garments. In particular, Acar et al. fabricated nylon graphene oxide (GO)-coated fibers, which were later embedded in an elastic armband; the evaluations on a single HC showed a 96% correlation between the ECG waveforms acquired with graphene textile electrodes and the conventional Ag/AgCl ones [22]. More accurate statistical data, on the other hand, were presented by Le et al. to compare the performances of silver-based textile electrodes (embedded in a smart bra) and Ag/AgCl gel counterparts [20]. A similar bra was designed and fabricated by Shathi et al. [27] which proved their reduced GO/poly(3,4-ethyelenedioxythiophne polystyrene sulfonate) (PEDOT:PSS) electrodes showed an improved ECG signal response in both wet and dry conditions; additionally, their e-textile electrodes demonstrated an improved flexibility, bendability, and stretchability when compared with that of conventional electrodes. The manufactured ones—integrated in the sports bra—were the final product of a fabrication study in which even other kinds of e-textile electrodes were analyzed [26]. Sinha et al. [16] fabricated and analyzed in the same period similar PEDOT:PSS-coated electrodes, demonstrating the capability of such devices to record ECG—and, in addition, electrodermal activity (EDA) and electromyography (EMG)—for a single HC in both dry and wet conditions.

Another interesting approach to fabricate e-textiles for ECG monitoring was proposed by Li et al. [18]. The authors designed and fabricated an e-textile solution combining the advantages of both the Japanese Kirigami pattering and the inkjet printing strategy demonstrating an ECG stable signal acquisition on a single HC with more than 100% strain of electrodes. Micro/nano fabrication strategies could be considered as a potential solution even for the fabrication of electrical interconnects/stretchable conductive adhesives (SCA). Specifically, a mixture, composed by Ag particles, MWCNTs and silicone rubber, was prepared and SCA electrodes were fabricated depositing such mixture on a polydimethylsiloxane (PDMS) layer, later integrated with a same layer deposited onto an elastic bandage [31]. From the outcomes of the investigations, the authors showed the ECG signal resulted both of good quality and thoroughly stable both for multiple patients’ configurations (even after the SCA-equipped elastic bandage was washed) if compared with the results obtained with commercial counterparts. Liang et al. [32] recently presented the preliminary results of a similar research. Specifically, they worked to develop a stable and biocompatible composite ink—a dispersion of silk sericine-MWCNTs—which could be processed using well-consolidated printing processes (e.g., inkjet printing). The authors demonstrated the ink could be used, even though a straightforward dying process, to fabricate conductive fibers/yarns and textiles “with desired mechanoelectrical properties” [32]. The integration of such conductive textiles (even breathable and reusable) on a compression shirt allowed to collect fine structures of the ECG signals in dry state, demonstrating the potential applicability of these smart clothes in healthcare (i.e., monitoring human biosignals). Another solution based on micro/nanofabrication strategies was proposed by Yao et al. [19] who manufactured silver nanowire/thermoplastic polyurethane (AgNW/TPU) electrodes to be later integrated on commercial patches. The authors demonstrated these devices were capable to acquire EGC—in dry state—of a quality comparable to the commercial gel electrodes; moreover, they did not find signal degradation up to 50% strain and 100 cycles. As shown in Table 2, in addition to ECG, even EMG and body motion signals were collected. Similar signals were analyzed even by Jin et al. [21] who used an e-textile sportswear in which an EMG sensor, a strain sensor, and a fluoroelastomer conductor, reinforced with polyvinylidene fluoride (PVDF) nanofibers, were integrated. The system showed the possibility to acquire ECG signal, and even the others, without significant degradation during a 1 h exercise of an HC.

In the last few years, although the research on the ECG monitoring in the field of wearable e-textiles seems still in a preliminary stage, few authors tried to develop slightly more complex e-textile solutions. For instance, in 2013 Kuroda et al. [13] proposed two prototypes of e-textile sensing vests, where different combinations of conductive and nonconductive yarns were investigated. The first prototype demonstrated the Japanese NISHIJIN production process was suitable to acquire a clean ECG signal as well as the second more advanced prototype (albeit fabricated—for an eventual mass production—using a different manufacturing technique), although some limitations in the ECG acquisition appeared [13]. In the same period, Catarino et al. [12] investigated the capabilities of a novel shirt prototype; specifically, three electrodes were knitted with Elitex for a double purpose: firstly, to allow the integration of electrical connections in the textile substrate, and secondly, to fix the electrodes in specific areas of the shirt prototype. Even if the ECG signals demonstrated different in case of either dry or wet electrodes, the authors claimed the results were of acceptable quality (considering conventional gel electrodes performances) and a tailor-made design of the shirt (according to the target patient) could potentially maximize the ECG acquisition performance [12]. Similar findings were presented even by Zięba et al. [29]. The authors showed a custom-made laminar textile electrode made of a silver woven fabric to be potentially integrated in a shirt and a sock, following predesigned configurations. The authors demonstrated (on a single HC) that the electroconductive material showed negligible difference with conventional electrodes and could be effectively used to potentially fabricate e-textile integrated shirts and/or socks. More recently, Tao et al. [30] integrated on a flexible printed circuit board (later, in turn, integrated on a sportswear shirt) an EGC sensor, connected, in turn, to a textile electrode by conductive threads. The authors validated the system demonstrating the effective ECG acquisition, even after 30 washing cycles when the print circuit board was integrated on a sportswear fabric using 4 mm thick PDMS.

The literature on e-textile applications for ECG acquisition showed even approaches for which the signal acquisition was only one of the milestones. For instance, Lopez et al. [24,25] designed and presented a medical Information Technology (IT) platform—based on multiple subsystems—for patients’ localization and monitoring. The authors proposed as a healthcare monitoring subsystem for ECG acquisition a Nylon/Lycra shirt into which two e-textile electrodes were integrated. The results—after acquiring ECG on 5 patients with cardiological diseases—showed ECG was of a higher quality (also in dry state) when the subjects were still, while the signal slightly worsened when sudden movements took place. However, the authors demonstrated the use of a conductive gel and/or mechanisms to reduce motion artifacts could improve signal quality [24]. Similar conclusions were presented further in their more recent article [25]. Similarly, Ferreira et al. [15] designed and presented the Baby Night Watch IT platform to monitor infants potentially affected by Sudden Infant Death Syndrome. In this study, a chest belt, into which electrodes and silver coated polyamide yarns were integrated, was chosen as healthcare monitoring subsystem, demonstrating a comparable performance with counterpart commercial products in terms of ECG measurements. In particular, both the custom-made and the commercial chest belts demonstrated capable to acquire very robust heart rate pulses when infants did not move and were lying on their back, while the authors found several missing heart beat pulses when infants were more active.

Finally, a few research groups also performed experiments on a relatively significant number of subjects (if compared to the already cited contributions). For instance, Postolache et al. [14] presented a wheelchair prototype where e-textiles, namely, electrodes made of fibers coated by conductive polymer and silver, were integrated in correspondence of the armrests. The data acquired by 7 HC demonstrated the proposed platform showed results comparable to that of the commercial counterparts. A similar number of HC were object of ECG acquisitions in the study of Arquilla et al. [28]. The authors manufactured a set of three electrodes—made of nylon coated by silver nanoparticles—stitched on a nonextensive fabric backing. Two minutes of ECG acquisitions on 8 HC demonstrated once again the capabilities of e-textile electrodes, showing their potential applicability across a wide range of anthropometries and skin types and signal invariance during stretch, bend, or wash tests. The most important diagnostic example, however, was such proposed by Fouassier et al. [17]. Specifically, the authors designed and manufactured a t-shirt prototype—into which electrodes made of silver yarns and hydrogel pads were integrated—aimed at working as a 12-lead ECG acquisition system. This solution allowed, to the best of the authors’ knowledge, short-duration 12-lead ECG acquisitions with quality levels comparable to conventional Holter recordings on 30 HC for 4 different analyzed positions.

Often, in the context of cardiac field, diagnostic data can be acquired also using simpler and/or different strategies. For instance, Lopez et al. [24,25] were also able to acquire simultaneously and show (on their IT platform) the hearth rate from 5 patients with cardiological diseases, using the same shirt used for ECG acquisition. Later, Dabby et al. [33] showed similar conclusions using another e-textile prototype; specifically, they demonstrated their e-textile solutions (bras, shirts, and shorts) demonstrated capable to acquire a heart rate signal comparable to a commercial chest strap. Equivalently, even Tao et al. [30] solution demonstrated capable to monitor the heart rate (calculated from the RR interval data of ECG signal).

Blood pulse, namely, pulse rate, is another potential diagnostic data that e-textiles can collect from patients. The first examples presented in this 10-year report of papers are those showed by Frydrysiak et al. [35,36]. The authors, partially inspired by their previous study [29], presented a solution to acquire the pulse signal from elderly people using a shirt into which the textile electrodes were integrated by different configurations. Analogously to Lopez et al. and Ferreira et al., the pulse acquisition was only one of the milestones of the presented textronics system, whose aim was even to collect other data (e.g., patients’ breathing rhythm) which are made available in real time for a potentially advanced subject monitoring. Quite recently, the research on blood pulse monitoring has gained increasing importance, as testified by the growing number of contributions appeared in the literature. For instance, Jang et al. [38] developed a composite fiber sensor which was later sewed in an electric armband applied, in turn, on an artificial arm with varied blood pressures. The experiment on this artificial setup demonstrated the system was capable to distinguish rachial artery pulses with varying blood pressure and pulse rate. Another recent solution was described in [37]. The authors presented a three-dimensional, composite spacer textile pressure/strain sensor, mainly designed for human motion detection purposes, which could even be used to monitor the arterial pulse pressure when the system was attached to one of the wrists of HCs. The results suggest the device could detect different pulse trends, which are linked to different pathologies and/or complications related to behavioral risk factors. Similar measurements were carried out by Shathi et al. [27], who acquired the pulse rate of a single HC demonstrating their e-textile electrode, in direct contact with the female volunteer’s wrist, showed a pulse response in nearly accordance with normal kits; some deflections/distortions in pulse rate were found during running. Similar results were presented even by Fan et al. [39]. They proposed a triboelectric all-textile sensor array (TATSA), a composite textile made of stainless steel fibers inserted into several pieces of one-ply Terylene yarns, which was conveniently embedded into several clothes such as wristbands, fingerstalls, socks, and chest straps. The TATSA proved not only to acquire a good quality, namely, in line with the signals acquired with other devices and solutions, pulse rate, but even the possibility to acquire and highlight differences in pulses depending on the measuring position (the authors applied TATSA on neck, wrist, fingertip, and ankle) and subjects’ ages, demonstrating the straightforward applicability of the solution to different populations. Simultaneously, Tang et al. [34] designed and manufactured a nonwoven fabric e-textile prototype, which demonstrated capable to effectively monitor blood pulse. Finally, in recent years, to the authors’ best knowledge, the last diagnostic solution in the field of cardiology, by means of e-textiles, was oriented to record lower extremity venous occlusion plethysmography (LEVOP). To this aim, Goy et al. [40] developed and fabricated a custom-made battery powered plethysmograph, connected on the one side to an oscilloscope, and on the other side on a set of different e-textile electrodes. The authors conducted LEVOP recordings on 5 HC demonstrating all the three set of the proposed e-textiles materials can be used for LEVOP recordings, showing additionally a statistical in-depth analysis related to the recorded signals from the different materials.

In conclusion, from the systematic analysis conducted, it was demonstrated the design and fabrication of e-textile solutions for biomedical applications gained considerable attention in recent years, and the promising results suggest a potential interest in further research. Maybe, to obtain a definitive answer regarding the direct practical applicability of these e-textile solutions, more studies involving larger cohort of subjects (healthy and pathological) are still required. However, as readers can ascertain reading the next subsections, currently this field seems to be the most advanced in this sense.

### 3.2. The Applications in the Muscular Setting

Surface Electromyography (sEMG) is a noninvasive methodology to measure muscle activity using surface electrodes placed on the skin overlying a muscle or a group of muscles [85]. This technique is widely used in rehabilitation research, sport sciences, kinesiology, and ergonomics [86]. Electrodes for sEMG are mostly combined with electrode gel to reduce the electrode-skin impedance [87]. Nevertheless, in recent decades, e-textile sensors, fabrics which are given sensing properties of different physical nature, such as capacitive, resistive, optical and solar, are increasingly spreading due to their wearable nature [88]. The researches in this field are summarized in Table 3. Ozturk and Yapici [43] proposed wearable graphene textile electrodes to monitor muscular activity showing their feasibility to acquire sEMG signals. They performed a benchmarking study with wet Ag/AgCl electrodes showing good agreement between the two technologies of electrodes in terms of signal-to-noise ratio (SNR) and signal morphology with correlation values up to 97% for sEMG signals acquired from the biceps brachii muscle. The same authors, in line with the previous conference paper [43], presented a research article [42] in which they underlined deeply the use of graphene-coated fabrics as textile electrodes in sEMG acquisition, considering not only the biceps brachii muscle but also triceps brachii and quadriceps femoris muscles. They performed a benchmarking study between the proposed textile electrodes and commercial wet Ag/AgCl ones for each muscle in terms of the skin-electrode impedance (SEI), SNR, and cross correlation reaching results within the range of commercial Ag/AgCl electrodes. The results demonstrated that graphene-coated textile fabrics could represent a valid alternative to gelled Ag/AgCl electrodes and therefore they could be used to develop wearable and smart garments. A similar work was conducted by Awan et al. [44] who investigated the use of a graphene-based electromyograph fabric sensor as a comparable alternative to commercial Ag/AgCl wet electrodes. The authors demonstrated that textile electrodes outperformed the standard Ag/AgCl electrodes in terms of SNR. Additionally, they, after tests on 8 HC, underlined graphene-based smart fabrics can potentially represent a viable alternative to non-reusable Ag/AgCl electrodes for high-quality EMG monitoring. Other authors proposed wearable devices to monitor EMG signals through textile electrodes; as first example, Nijima et al. [46] proposed a wearable EMG sensor for monitoring masticatory muscles with PEDOT:PSS textile electrodes with the aim to monitor daily activities such as diet, sleep bruxism, and human motor control. The same authors in a more recent work [45] used the above-mentioned prototype to monitor muscle fatigue related to the muscles of the limbs, starting from the acquisition of temporal muscles, based on the assumption that there is a strong correlation between frowning and jaw clenching muscle activity and the physical efforts made when exercising. Choudhry et al. [48] designed textile-based piezoresistive sensors developed using flexible conductive threads stitched on fabric. They embedded the sensor inside a garment to measure small pressure changes exerted by human muscles. Other authors proposed multifunctional e-textiles to monitor several vital signals, EMG signals included. As described in Section 3.1, Yao et al. [19] developed an integrated textile patch comprising four dry electrophysiological electrodes, a capacitive strain sensor, and a wireless heater for electrophysiological monitoring, motion tracking, and thermotherapy, respectively. Jin et al. [21] showed their solution demonstrated its feasibility for continuous long-term monitoring of ECG, EMG signal and motion during 1 h of weight-lifting excercises without significant degradation of signal quality. As third example, Sinha et al. [16] showed how PEDOT:PSS coated electrodes, integrated in a spandex t-shirt, were effectively able to record simultaneously EMG, ECG, and EDA in dry state. The authors concluded this solution could represent a tool for continuous and simultaneous measurement of vital signals in at-risk patients. Samy et al. [41] employed five EMG electrodes: three were attached to subject’s chin to detect its muscle movement, which can be indicative of teeth grinding (bruxism), sleep apnea, and other sleep disorders, while the other two electrodes were attached to the legs, between the knee and the ankle, to record leg movement. Finally, Farina et al. [47] proposed the use of Smart Fabric and Interactive Textile system as an alternative solution for recording high-density EMG signals for myoelectric control. They designed a sleeve covering the upper and lower arm containing 100 electrodes arranged in four grids of 5 × 5 electrodes for EMG. The textile electrodes were realized with stainless steel yarns and they had a diameter of 10 mm and an interelectrode distance of 20 mm. The proposed method for interfacing myoelectric prostheses with the neuromuscular system by integrating electrodes in garments proved its feasibility, allowing for high accuracy in EMG classification.

From the analysis carried out on this topic it is possible to conclude that several technologies and materials were proposed for the realization of electrodes in e-textile able to acquire EMG signals. Future investigation on enriched study population both normal and pathological will confirm the potential the utility of textile electrodes in clinical practice to replace well-known pregelled electrodes.

### 3.3. The Applications in Orthopaedics

Recently, the development and the spread of Inertial Measurement Units (IMUs) for spatiotemporal and kinematic assessment has represented an innovative progress in the field of biomechanics and wearable sensors. Indeed, wearable sensors based on IMUs are spreading in the biomedical field showing good performances [89,90,91] compared to their gold standards. Moreover, considering that the working principle of IMUs is based on the measurement of inertia, IMUs can be applied anywhere without a reference [92] and integrated with textile technology [93]. The research in this field is summarized in Table 4. Bartalesi et al. [53], indeed, developed a wearable system that integrates and fuses information gathered from textile-based piezoresistive sensor arrays and triaxial accelerometers, which demonstrated able to perform a real time estimation of the local curvature and the length of the spine lumbar arch. The authors performed a comparative study between their system and a stereophotogrammetric system, showing a very low error when reconstructing the lumbar arch length of a single HC. Considering the same idea (namely, merging several technologies), Li et al. [58] presented a method to integrate and package a triaxial accelerometer within a textile as to create an e-textile fully integrated within the weave structure of the fabric itself, making it invisible to the wearer. The integrated e-textile based accelerometer sensor system placed on arm and knee joints was used to identify the activity type, such as walking or running, through the calculation of the joint bending angles. They performed a benchmarking analysis between the proposed device and the related gold standard, showing good agreement with an error lower than 1%. Amitrano et al. [61] proposed a new wearable e-textile based system for biomedical signals remote monitoring able to acquire angular velocities of the lower limbs. Specifically, the system equips an IMU and textile pressure sensors made of EeonTex, namely a conductive and nonwoven microfiber with piezo-resistive functionality, which were placed correspondingly between the ankle and the plantar zone of the considered (3) HCs, respectively. The proposed system finds wide application in the field of remote monitoring and telemedicine.

Other research groups proposed wearable systems for remote monitoring; for instance, Lorussi et al. [60] proposed a wearable system to remotely monitor musculoskeletal disorders. The system is composed of IMUs, e-textile sensors and a decision support system included in a dedicated app able to assist the patient in performing personalized rehabilitation exercises designed by a physician/therapist, remotely and in real-time (also through alerts). Raad et al. [55] proposed a wearable smart glove for remote monitoring of rheumatoid arthritis patients monitoring finger flexions while patients performed several activities at home. The e-textile glove used flex and force sensors and an Arduino platform to transmit motion data to the physiotherapists through a mobile phone, on which a dedicated app is installed. Other authors proposed a complete platform for healthcare monitoring. As described in Section 3.1, Lopez et al. [25] proposed a novel healthcare IT platform capable of monitoring several physiological parameters, such as electrocardiogram (ECG), heart rate, body temperature, and the capability to track the location of a group of patients within hospital environments through the combination of e-textiles and wireless sensors. The same authors, in another work [24], proposed a medical IT platform, based on wireless sensor networks and e-textiles, which supports indoor location-aware services as well as monitors physiological parameters, such as ECG, heart rate, and body temperature. Tao et al. proposed a totally flexible and washable textronic device able to acquire several types of biological data. The data containing vital physiological signs, skin temperature, and activity motions were transferred via low-energy Bluetooth technology to a smart phone and then via 4G or Wi-Fi into a remote data server to realize a continuously Web-based monitoring system. Other researchers proposed wearable devices that are completely textile and do not integrate devices such as IMUs. In this context, Della Toffola et al. [54] proposed a wearable system for long-term monitoring of knee kinematics: compliance with the use of knee sleeve is monitored by using an e-textile sensor that measures the knee sleeve fabric stretch, thus allowing to infer whether the subjects under test wears the knee sleeve. Garcia Patino et al. [63] proposed a compact textile-based wearable platform to track trunk movements when the considered user bends forward. The smart garment developed for this purpose was prototyped with an inductive sensor formed by sewing a copper wire into an elastic fabric in a zigzag pattern. Heo et al. [50] proposed a flexible glove sensor—which included stretchable and flexible PDMS films—to monitor upper extremity prosthesis functions. Other researchers studied new arrangements of materials for biomedical applications, Jin et al. [21] studied a highly durable nanofiber-reinforced metal elastomer composite consisting of metal fillers, an elastomeric binder matrix, and electro-spun PVDF nanofibers to enhance both cyclic stability and conductivity, showing a good continuous long-term monitoring of ECG, EMG signal, and motions during weightlifting exercises without significant degradation of signal quality. Li et al. [64] fabricated a textile-based stretchable sensor by using an electronic dyeing method; the conductive textile showed good flexibility and adaptable strain-electric response. The authors demonstrated the excellent performances for monitoring and analysis of several human activities. Tang et al. [34] reported the functionalized conductive, sensitive, wearable, and washable vacuum pressure sensor based on carbon nanotubes (CNTs), e-textile with unique nanostructures growth on the non-woven fabric by using the novel, and facile nano-soldering method. They proved that CNTs e-textile sensor has a good linearity, high sensitivity and low power consumption. Moreover, they showed the good repeatability, washability, durability, and super-hydrophobic performance of the CNTs underling its feasibility to realize smart clothes. Yao et al. [19] presented mechanically and electrically robust integration of nanocomposites with textiles by laser scribing and heat press lamination showing a good washability and good electromechanical performance up to 50% strain. They underlined the potential utility of these new materials and methods in healthcare, activity tracking, rehabilitation, sports, medicine, and human-machine interactions. Finally, Ye et al. [51] reported a scalable dip-coating strategy to construct conductive silk fibers showing their feasibility to be woven into fabrics, resulting in textiles sensitive to physical stimuli such as: force, strain, and temperature.

Park et al. [65] proposed a dynamically stretchable high-performance supercapacitor fabricated with MWCNT/MoO3 for powering an integrated sensor in an all-in-one textile system to detect various bio signals. This system sewed into cloth both t-shirt and glove successfully detects strain due to joint movement and the wrist pulse.

Zhang et al. [57] designed a fabric E-textile for tracking active motion signals. The fiber-shaped coaxial tribo-sensor is fabricated with silver yarn and polytetrafluoroethylene yarn, which allows for integrating well with cloths at large scales due to its satisfactory breathability, good washability, and desirable flexibility.

Jang et al. [38] proposed a highly sensitive fiber-type strain sensor with a broad range of strain by introducing a single active layer onto the fiber. The sensors were sewn into electrical fabric bands, which are integrable to a wireless transmitter to monitor waveforms of pulsations, respirations, and various postures of level of bending a spinal cord. About the last issue, the authors developed an electronic band-type posture corrector (E-posture-corrector) with the fiber sensor to continuously measure resistive changes to bending angles of a human spinal cord.

Kiaghadi et al. [59] presented the design of Tribexor, an end-to-end sensing system that leverages triboelectric textiles to measure joint motions and sweating behavior showing that the sensor has high performance in natural conditions by benchmarking the accuracy of sensing several kinematic metrics as well as sweat level.

Other authors focused on activity recognition and monitoring. Fevgas et al. [52], indeed, presented a platform and a methodology for rapid prototype development of e-textile applications for human activity monitoring to address the problems of human movement and gesture monitoring, posture recognition and fall detection. Kim et al. [37] proposed a carbon nanotube ink drop-coated textile resistive pressure sensor on a typical three-dimensional spacer textile able to detect human health and motion. The resulting 3D spacer textile pressure sensor unit showed a wide range of sensing performance of 200 kPa–50 kPa, which facilitates the detection of physiological signals, such as acoustic vibrations and hand motion. Vu et al. [56] introduced a new approach to classify human body movements, by using textile sensors, embedded into fabrics, using AI to recognize different standard human motions (e.g., walking, jumping, running, and sprinting) starting from features extracted from strain signals. The last authors proposed also another work [49] in which they presented an e-textile strain sensor integrated on a glove to monitor angles of finger motions. They also proved the feasibility of this sensor placing it onto the skin of the neck to record the pharynx motions when speaking, coughing and swallowing. Samy et al. [41] proposed an unobtrusive framework for sleep stage identification based on a high-resolution, pressure-sensitive e-textile bed sheet able to acquire information related to body movement, posture, and body orientation. Finally, Hayashi et al. [62] proposed a smart wheelchair, composed of e-textile pressure sensors placed on the seat and back support, able to monitor the patients posture on the basis of quantitative sitting-posture scores.

The articles related to this section show how the integration of IMUs for movement assessment in e-textile garments is a consolidated practice. By means of the integration of algorithms, it is possible to compute several kinematic parameters starting from acceleration or angular velocities. Moreover, the kinematic information can be acquired thanks to the capability of some materials described in some articles to change their intrinsic resistance when they are stretched showing their feasibility in the movement assessment.

### 3.4. The Applications in the Respiratory Tract

Respiration is a crucial vital function for humans; abnormalities in such a function may have a different origin and can lead to patient deterioration and, ultimately, death. Previous research extensively documented the clinical importance of respiratory rate diagnosis and how precise and routine monitoring is yet to be achieved, on the one side due to intrinsic difficulties (linked both to human and machines limitations) and on the other side because of limited use and/or the small diffusion of advanced respiratory monitoring systems [94,95]. To reach this gap, several methods were proposed and were widely investigated over time [96,97]; among these, several e-textile applications were also proposed. The research in this field is summarized in Table 5.

To the best of the authors’ knowledge, Zięba et al. [68] presented the first prototype in this 10-year report of papers. The proposed solution was a textronic shirt with sensorial stripes (namely, a textile knitted sensor made of silver yarns) whose ends were connected to a current amplifier to acquire breathing rhythm. The authors evidenced the device was able to acquire the signal, although some limitations (e.g., sensors positioning) could not be overlooked when the HC was not at rest during the acquisitions. In the same period, the authors [69] presented a similar prototype with a different configuration—and amount—of the sensorial stripes (in this case, the authors embedded 3 stripes upon the chest). Correspondingly, a similar but different prototype—namely, the same textronic shirt equipped with two sensorial stripes localized upon the chest—was proposed to monitor the elderlies’ breathing rhythm [35,36]. In the same period , Ramos–Garcia et al. designed and fabricated a Respiratory Inductive Plethysmograph based breathing system aimed at potentially monitoring breathing rate. The proposed system was a polyester/spandex t-shirt on which a stretch e-textile sensor was placed around the HC’ chest. The preliminary results indicated the proposed system, which needs further improvements to be properly used for multiple tasks, was capable of effectively monitoring breathing rate of 3 HCs. Another more recent solution was described in [30]. The authors used the same sportswear shirt prototype—already described in Section 3.1 for the ECG acquisition—to detect the respiration rate using the same ECG sensor connected to a different textile electrode. The more recent example in this 10-year report of papers is, to our best knowledge, the solution adopted by Fan et al. [39], who positioned the TATSA around the chest to monitor the respiratory signal. Summing up the promising results related to both the cardiac and the respiratory signals, the authors proceeded even with new experiments assessing a different shirt configuration (designed by embedding two TATSA on the abdomen and wrist positions to monitor both the respiratory and pulse signal, respectively) which allowed to effectively ascertain respiratory—and, as expected, heart rate-related variations—differences in an HC and a patient affected by sleep apnea syndrome.

Another solution to potentially monitor this pathology using e-textile prototypes was described in [66]. Specifically, the authors manufactured an e-textile bed sheet (where the e-textile piezoresistive layer is enclosed between two sheets of conventional fabric layers) aimed to indirectly acquire respiratory rate data; additionally, the authors designed an IT platform capable of accurately (yet noninvasively) processing the acquired data. The analyses performed on 14 HC demonstrated that the overall system was effectively capable to inconspicuously monitor patients’ respiratory rate when they slept in supine position. Albeit patients’ movements effectively invalidated respiratory rate monitoring, the system demonstrated a valid tool to track diseases (e.g., the already mentioned apnea disease) for which patients movements can be limited. Similar e-textiles and IT platforms were used later from the same research group and other colleagues. On the one hand, Liu et al. [67] conducted new investigations to automatically monitor respiratory rate, considering either the analysis of a restricted patient area (e.g., torso) or different bed configurations (e.g., tilted bed setups); on the other hand, Samy et al. [41] concentrated, as already described in Section 3.2, on new objectives, among which we can mention the sleep stage analysis. In this case, the respiratory rate (even when acquired by the e-textile bedsheet) demonstrated a different output during the different sleep stages of the patients. This finding can help to design and implement the proposed device as an unobtrusive sleep stage identification system, which would help to potentially perform early diagnoses of sleep disorders and chronic diseases. Respiratory rate demonstrated important even in the case of infants sleeping monitoring [66]. It is not by chance if Ferreira et al. [15] investigated—using their custom-made chest belt and the Baby Night Watch IT platform (see also Section 3.1)—respiratory rate variations in infants to monitor eventual Sudden Infant Death Syndrome events; nevertheless, albeit the chest belt represents for all intents and purposes an e-textile system, respiratory rate was acquired by the authors using a triaxial accelerometer integrated in the chest belt, differently from the ECG signal. An e-textile embedded chest belt was developed even by Jang et al. [38]. Specifically, the authors sewed horizontally on the chest belt the same composite fiber sensor (developed even for pulse acquisition), aiming at monitoring respiratory waveforms from an HC in various breathing conditions. The authors claimed the results agree with those found using a commercial breathing sensor and, finally, hypothesized the fiber sensor could be potentially used to fabricate devices capable to evaluate the breathing quantities (for instance, respiration volume, and lung capacities). Another recent e-textile prototype for the potential diagnosis of respiratory signal related diseases was that presented by Choudhry et al. [48], who integrated on a vest a different multilayer sensor around a HC’s ribcage area. The preliminary results indicated the system was capable to recognize indirect changes in breath pressure and that the acquired signal was coherent with average adult breaths count. Finally, Lian et al. [70] proposed a multifunctional e-textile material—whose layers were composed of high-density AgNWs and a sensing fabric, respectively—which was used to fabricate a face mask, through which they showed the feasibility to indirectly evaluate variations in breathing rate. This prototype could show potential applications also for healthcare monitoring (e.g., cardiac and respiratory illnesses linked to particulate matter 2.5 penetration in human body); however, to the best of the authors’ knowledge, the authors did not consider this particular case as the main application for the multifunctional e-textile. Differently, in the same period another prototype of e-textile mask was presented by Liang et al. [32]. Contrarily from the previous paper, in this case the authors merely sewed the conductive textiles (briefly described in Section 3.1 yet) onto a mask which was capable, thanks to the sensibility of the conductive textile components to ascertain humidity variations which are different during HCs’ periodic exhaling and inhaling, of precisely monitoring HCs’ breath, which can help to keep under control or avoid pathologies such as lethal sleep apnea.

From the outcome of our investigation it is possible to conclude that, as already seen for the previous subsections, further research is needed validate the direct practical applicability of these e-textile solutions. Nevertheless, since a lower number of publications on this topic—probably a consequence of a scarce research interest by many research group—seem to be available in the literature, the “bench to the bedside” process (and, consequently, the needed temporal range) would be more complicated than the solutions considered in the previously analyzed fields.

### 3.5. Miscellaneous

The previous subsections have dealt with the main themes on which the applications and developments of e-textile technologies focused. However, there are applications of e-textile even on less common themes, which confirm the wide development of these technologies in the last decade and testify to the variety of purposes to which textile technologies can be applied. In this paragraph, we collected several works that offer applications on ’other themes’ different from those described in detail in the previous subsections, widening the horizon of biomedical applications of e-textile technologies. The research presented in this subsection is summarized in Table 6.

Golparvar and Yapici focused their work on the use of e-textiles in the field of electrooculography (EOG), proposing, for the first time, the use of graphene-coated fabric electrodes for electrooculogram acquisition. In [72] they performed a comparative study between conventional Ag/AgCl electrodes and their e-textile electrodes demonstrating high degree of flexibility, elasticity, and the possibility of incorporating the novel electrodes into various types of personal clothing. The following year, the same authors presented two research articles [73,74] in which they designed a devoted unit for textile-based EOG that can achieve on-board noise removal and signal amplification. Moreover, they developed and implemented a controlled automatic blink detection algorithm, able to detect voluntary blinks in real-time. The performances of the device in recording EOG signal during specific eye movement patterns and in detecting voluntary blinks were explored in their works resulting in good agreement with the reference EOG systems based on Ag/AgCl electrodes.

EDA, also known as galvanic skin response (GSR), is another bioelectrical signal, usually recorded with common Ag/AgCl electrodes, which was one of the subject of study and applications of textile-based electrodes and devices. Sinha et al. [16] employed the same PEDOT:PSS based electrodes used for ECG and EMG recording even to collect EDA signal from fingers and wrist. To this aim, they developed a sensing shirt able to simultaneously record the three biosignals, finding potential applications in continuous health monitoring as well as physiotherapy. Similarly, Postolache et al. [14] developed e-textile electrodes for measuring skin conductance using the same materials employed for ECG recording (textile made of fibers coated with conductive polymer and silver). E-textile electrodes were attached to the wheelchair armrests to monitor physiological stress parameters of the wheelchair user in unobtrusive way. Haddad et al. [75] used a different approach to develop EDA electrodes; specifically, they integrated Ag/AgCl uniformly coated yarns within three different textile substrates (100% cotton, 100% nylon, and 100% polyester). The e-textile electrodes were used to record EDA on the distal phalanx of the fingers, and their performances were compared with the standard rigid Ag/AgCl electrodes, resulting in higher stability for e-textile electrodes when changes in skin temperature occurred. Jennifer Healey [76] proposed a different application of GSR measurement, developing a ‘GSR sock’ by integrating two fabric electrodes from a commercial heart rate monitor strap into a standard sock. The electrodes were placed to make contact with the ball and heel foot of a HC. The experimental testing showed that the sock prototype provided a meaningful measure of GSR activity that can be used unobtrusively in daily monitoring.

Chen et al. [81] applied their expertise in flexible electronics and polymers to develop a fiber-shaped e-textile strain sensor using polyurethane fibers, AgNWs and styrene-butadiene-styrene (SBS) via knitting and simple dip-coating processes. Due to the textile-based structures and hierarchical fibers, the e-textile exhibited good capability of detecting multiple deformation, including tensile strain and pressure, which enables a wide range of biomedical purposes. In particular, the authors proposed different applications in health monitoring such as pulse beating detection, phonation detection, scoliosis correction, and Restless Legs Syndrome (RLS) diagnosis. A similar strain sensor was developed by Vu & Kim [49], using a slightly different manufacturing process: a polyester/spandex fabric was immersed in a single-walled carbon nanotubes (SWCNT) ink, which gives sensing capabilities, and, after squeezing and drying processes, silver pastes were printed onto the SWCNT fabric to improve strain sensor performance. Within the manuscript, the authors provided an extensive characterization of the properties of the fabric, testing sensors with different shapes and structures. To demonstrate the potential of their sensors in practical applications, Vu & Kim proposed to attach the textile sensor to the skin of the neck, to monitoring pharynx motion, demonstrating that is possible to obtain consistent signals when speaking, coughing, and swallowing. Before them, Kim et al. [37] already explored the possibility of registering the movement of the pharynx with a textile sensor. They used a carbon nanotube ink drop-coated textile resistive sensor on a three-dimensional spacer structure, to monitor pharynx movement during speaking. In their experimental testing, authors demonstrated that the recorded signals exhibited distinct profiles when different words are pronounced, and the same word generated a similar wave profile in repeated tests. The same sensor was also tested attached to the cheek skin, demonstrating the ability to detect cheek bulging movements. Following these results, authors pointed out the potential applications of the sensor in human-machine interaction and as a face and speech recognition system.

Another important biomedical application, which is particularly suitable with wearable and textile-based electronics, is the measure of skin temperature. This is an important parameter for a variety of health monitoring applications, where changes in temperature can indicate changes in health. Embedding temperature sensors within textiles provides an easy method for directly measuring body temperature in defined areas. This is why many researchers, even though they do not have a focus in temperature-related health effects, integrated a temperature sensor into the devices they developed to add an additional information to the recorded health data. As first example, Lopez et al. [24,25] embedded a thermometer in the Wearable Data Acquisition Device to include the body temperature as a further parameter provided by the proposed healthcare monitoring system. Similarly, Tao et al. [30] exploited the temperature sensor integrated into the MEMS sensor chip they used for activity recognition purposes, to monitor user skin temperature. Ferreira et al. [15] used an infrared thermopile sensor embedded in the wearable chest belt, to measure the body temperature of infants, adding this parameter to the other signals registered by the device and previously discussed in the subsections of this Review. Other examples are provided by the two works by Frydisiak & Tesiorowski [35,36]. They developed a system to remotely monitor elderly people by acquiring various physiological parameters including, in addition to those already mentioned in the previous sections, underclothing temperature. Moving to more specific textile-based sensors, Lugoda et al. [77] developed a temperature sensing yarn, using a micro thermistor covered with packing fibers and a warp knitted tube. The temperature sensing yarns were then used to create a series of temperature sensing garments: armbands, a glove, and a sock. The performances of the temperature sensing wearable devices were investigated and, from the outcomes of the analyses conducted, the authors found some limitations in measuring skin temperature due to the deformation of the yarn structure and also depending on the fit of the garment.

Jiang et al. [78] also proposed a wearable sensing device with embedded temperature and humidity sensors, the latter used as sweat sensor. However, the authors focused on the use of a textile based Near-Field Communication (NFC) antenna, which is able to power the system and transmit sensors data. The measurement results have shown that the textile NFC antennas can still perform properly under bending up to 150°, with a maximum range of 6 cm to access sensor data. This innovation figures to be a very attractive field of development towards self-powered wearable devices, to overcome the limitations of power supplies, very critical challenges for the e-textile field. Sweat volume monitoring on skin is also one of the topics of the work by Kiaghadi et al. [59]. Authors designed a triboelectric textile sensor to measure joint movement, but they noticed that the baseline signal varied due to sweat volume produced on the skin. The reason is that the sweat induces the wetting of the inner layers of the sensor, whereas the outer layers, that are close to air, remain drier. This results in a different impedance between the layers, causing a small DC offset that is amplified in the electronics circuits, creating an observable change at the output of the textile sensor. The baseline changes thus reflected the sweat volume production on the skin. The high performance of the proposed sensor were also demonstrated in real-world applications, by benchmarking its robustness in perspiration measurements during exercise, comparing the results with those of a GSR sensor for skin conductivity.

Many researchers also proposed sensor to analyze the chemical composition of sweat, to investigate its constituents, which could be related to the subject’s physiological condition. Lactate and sodium are commonly analyzed markers in sweat, directly measured on body and, thus, very suitable for wearable textile application. On this research topic, Zhao et al. [80] presented a thread-based wearable biosensor to simultaneously measure concentration of lactate and sodium in sweat. To assess the performance of this wearable nanobiosensor, the authors developed an integrated smart headband to acquire data directly on the body. Tests were performed on a male volunteer subject during intense workout, and the sweat concentration was compared with the results obtained with standard methods, confirming the accuracy and stability of the biosensor in real use. Another electrochemical textile sensor was recently proposed by Liang et al. [32]. Fabricated via stencil printing Silk-Sericine Carbon Nanotube (SSCNT) ink and silver chloride paste on a PET film, the electrochemical sensor is capable to detect the concentration of hydrogen peroxide, which is an important intermediate in biological processes. The sensing mechanism is based on the electrocatalytic activity and conductivity of CNTs, very responsive to the change of concentration of hydrogen peroxide.

A very interesting application of textile electronics was presented by Mason et al. [79]. The authors investigated the response of a smart fabric, with integrated conductive pathways, strain gauges, and conductive pressure sensor points, at microwave frequencies region for data transmission of biomedical signals. The aim of this perspective research is to demonstrate the feasibility of the proposed smart sensing garments to detect dielectric changes directly on body. It is also shown how these sensing features can be exploited to monitor biomedical signals such as ECG and EMG, body temperature, sweat volume, etc. This novel sensor was even patented owing to the great potentialities shown in biomedical applications.

Finally, Rong Liu et al. [82] presented a peculiar application of e-textile, developing intelligent pants for monitoring incontinence status. The smart garment was developed incorporating conductive yarns in fabrics, using advanced circular seamless knitting technology. The presence of urine causes the variation of the measured electrical resistance of the conductive pathways, allowing to sense, monitor, and alert wearers and care providers on urinary incontinence status.

The overview proposed in this section demonstrates the wide range of biomedical applications that e-textile technologies can cover. The interest of researchers is not limited to the fields of medicine that are more easily explored by textile solutions, but also extends to apparently minor applications that, as demonstrated, can be developed and have great employment opportunities. In the works analyzed in this section, the focus is mainly on the development of new materials, which are sensitive to a certain physical quantity without losing the biocompatibility features. However, this prevailing focus on the development of the textile element means that less effort is invested in the clinical application of these products. For this reason, even in this case, the experimental procedures proposed are limited to proofs of concept on a limited cohort of healthy controls, while a structured clinical trial is never reported.

## 4. Conclusions

This systematic review showed the development of e-textile applications in the medical field for a decade. Several specialties of medicine were analyzed in these years: cardiology, a particular emphasis on muscles, physiatry and orthopaedics, respiratory tract, and also sparse studies on other themes were found. The studies are variegated in purposes but there is one big common limitation that comes out from this review: most of the studies focused on the development and testing of new devices on a single healthy subject, and only few studies considered a dataset made of more than 10 s of HC. Therefore, researchers should consider validating their novel devices on a larger cohort of subjects (healthy and pathological) for further studies. Many of the analyzed studies did not even mention the number of subjects tested as a limitation for their research. Perhaps this is because the development of these technologies is still in an early phase and the aim of the researchers was to improve the technology itself, leading up to the potential future goal of an experimental campaign on larger datasets. Following this brief discussion, our review should help researchers understand that it is now the time fora second phase, in which the devices are tested on larger datasets.

## Figures and Tables

**Table 1:** Number of instances for each acquired data, data type, and potential diagnosis combined with related references.

Biomedical Field	Acquired Data	Instances
Cardiac	ECG	21 [12,13,14,15,16,17,18,19,20,21,22,23,24,25,26,27,28,29,30,31,32]
	Heart rate	3 [24,25,33]
	Blood pulse	7 [27,34,35,36,37,38,39]
	LEVOP	1 [40]
Muscular	EMG	10 [16,19,21,41,42,43,44,45,46,47]
	Pressure signal from muscles	1 [48]
Physiatry/Orthopaedics	Finger flexion angles	4 [37,49,50,51]
	Acceleration data	4 [30,52,53,54]
	Angle of inclination	2 [24,25]
	Motion signals	6 [19,21,34,55,56,57]
	Elbow flexion angles	2 [58,59]
	Knee flexion angle	3 [51,58,60]
	Scapular flexion angles	1 [60]
	Angular velocity signal	1 [61]
	Plantar pressures	1 [61]
	Sleep posture	1 [41]
	FS and LL indexes	1 [62]
	Back movements	1 [63]
	Spinal cord bending angles	1 [38]
	Strain signals	4 [53,54,64,65]
Respiratory	Respiratory rate	7 [15,30,41,66,67,68,69]
	Breath pressure	2 [48,70]
	Breath signal	6 [32,35,36,38,39,71]
Other Themes	EOG	3 [72,73,74]
	EDA	4 [14,16,75,76]
	Skin temperature	8 [15,24,25,30,35,36,77,78]
	Biomedical microwave sensing	1 [79]
	Pharynx motion	2 [37,49]
	Cheek motion	1 [37]
	Sodium and lactate concentration in human sweat	1 [80]
	Sweat Volume	2 [59,78]
	Resistance signals	1 [81]
	Alert of the volume of leaked urine	1 [82]
	Hydrogen peroxide concentration	1 [32]

**Abbreviations**. ECG: Electrocardiogram; EDA: Electrodermal Activity; EMG: Electromyography; EOG: Electrooculography; FS: Forward Shift; LEVOP: Lower Extremity Venous Occlusion Plethysmography; LL: Lateral Lean.

**Table 2:** Insights regarding cardiac literature: authors, aim, dataset, and acquired data.

Authors	Aim	Dataset	Acquired Data
Lopez et al. (2010a) [24]	Describing a novel healthcare IT platform for localization and monitoring within hospital environments	5 PP	ECG; Heart rate; Angle of inclination; Activity index; Body temperature; Patient’s location; Battery level; Alert code
Lopez et al. (2010b) [25]	Presenting a medical IT platform platform based on Wireless Sensor Networks and e-textile for patients’ localization and monitoring	5 PP	ECG; Heart rate; Angle of inclination; Activity index; Body temperature; Patient’s location; Battery level; Alert code
Wu et al. (2010) [23]	Presenting a novel cloth electrode for ECG monitoring	1 HC	ECG
Zieba et al. (2011) [29]	Creating new sensorical clothing structures to measure human physiological signals in a non-invasive way	1 HC	ECG
Catarino et al. (2012) [12]	Designing and fabricating textile integrated electrodes for ECG continuous health monitoring for disabled or elderly people	1 HC	ECG
Kuroda et al. (2013) [13]	Prototyping an ECG sensing e-textile vest	1 HC	ECG
Goy et al. (2013) [40]	Fabricating e-textiles to monitor LEVOP	5 HC	LEVOP
Postolache et al. (2014) [14]	Presenting a wheelchair architecture equipped with e-textiles for ECG and SKC sensing	7 HC	ECG; EDA
Ferreira et al. (2016) [15]	Presenting the design and fabrication of SWSs to prevent infants’ SIDS	HC ^#^	Body temperature; Respiratory rate; ECG
Frydisiak & Tesiorowski (2016a) [35]	Designing a smart textronic shirts for the health monitoring of elderly people	HC ^#^	Blood Pulse; Breath Signal; Skin Temperature
Frydisiak & Tesiorowski (2016b) [36]	Designing a smart textronic shirts for the health monitoring of elderly people	HC ^#^	Blood Pulse; Breath Signal; Skin Temperature
Dabby et al. (2017) [33]	Presenting a new method for building wearable electronic and textile sensor systems directly integrated in garments to detect the heart rate	1 HC	Heart Rate
Acar et al. (2018) [22]	Developing a single-arm ECG armband embedded with flexible graphene textiles for ECG data acquisition	1 HC	ECG
Tao et al. (2018) [30]	Presenting a novel system—made up of a washable and wearable smart textile shirt, smartphone app and software desktop—for the acquisition of ECG signal, breathing rate, acceleration data for activity recognition and skin temperature	5 HC ${}^{ML}$ HC ^#^	ECG; Skin temperature; Respiratory rate; Acceleration data
Li et al. (2019) [18]	Fabricating e-textiles depositing conducting materials thorough inkjet printing on conventional textiles for monitoring purposes	1 HC	ECG
Yao et al. (2019) [19]	Designing and fabricating multifunctional e-textiles with mechanical and functional properties comparable with typical textiles for monitoring applications	1 HC	ECG; EMG (arm); Motion signals
Le et al. (2019) [20]	Comparing differences in ECG registration between silver-based textile electrodes and silver/silver-chloride gel electrodes, both integrated in a smart bra	1 HC	ECG
Jin et al. (2019) [21]	Fabricating a metal–elastomer–nanofibers conductive material for long-term monitoring	1 HC	ECG; EMG (bicep muscle); Motion signals
Kim et al. (2019) [37]	Developing an all-textile based pressure/strain sensor for physiological signals using 3D spacer textile	HC ^#^	Blood Pulse (wrist and neck); Finger flexion angles; Cheek motion; Pharynx motion
Ko et al. (2019) [31]	Designing SCAs for various applications	1 HC	ECG
Jang et al. (2019) [38]	Preparing a highly sensitive fiber-type strain sensor with a broad range of strain by introducing a single active layer onto the fiber	1 HC	Blood Pulse; Spinal Cord Bending Angles; Breath Signal
Fouassier et al. (2019) [17]	Comparing the quality of the ECG signal registered using both a 12-lead Holter and a novel smart 12-lead ECG acquisition T-shirt	30 HC	ECG
Sinha et al. (2020) [16]	Fabricating PEDOT:PSS coated electrodes to record EMG, ECG and EDA	4 HC ${}^{emg}$ 1 HC ${}^{eda}$ 1 HC ${}^{ecg}$	EDA; ECG; EMG (biceps, triceps, tibialis, and quadriceps)
Tang et al. (2020) [34]	Fabricating machine-washable e-textiles with high strain sensitivity and high thermal conduction for monitoring applications	1 HC	Motion signals; Blood pulse
Arquilla et al. (2020) [28]	Using sewn textile electrodes for ECG monitoring	8 HC	ECG
Shathi et al. (2020a) [26]	Presenting a highly flexible and wearable e-textile for smart clothing and ECG detection	1 HC	ECG
Shathi et al. (2020b) [27]	Developing e-textile electrodes for the detection of high-quality biomedical signals	1 HC	ECG; Blood pulse
Liang et al. (2020) [32]	Developing a stable and biocompatible silk sericine carbon nanotubes (CNT) ink and demonstrating its versatile applications in flexible electronics for monitoring human biosignals	HC ^#^	ECG, Breath Signal; Hydrogen peroxide concentration
Fan et al. (2020) [39]	Developing TATSA for precise epidermal physiological signal monitoring	1 HC 1 PP	Blood Pulse; Breath Signal

^#^ number of subjects not provided; ^*ecg*^: Electrocardiographic acquisitions; ^*eda*^: Electrodermal Activity acquisitions; ^*emg*^: Electromyographic acquisitions; ^*ML*^: Machine Learning training set. **Abbreviations**. ECG: Electrocardiogram; EDA: Electrodermal Activity; EMG: Electromyography; HC: Healthy Controls; IT: Information Technology; LEVOP: Lower Extremity Venous Occlusion Plethysmography; PEDOT:PSS: Poly(3,4-Ethyelenedioxythiophne) Polystyrene Sulfonate); PP: Pathological Patients; SCAs: Stretchable Conductive Adhesives; SIDS: Sudden Infant Death Syndrome; SKC: Skin Conductivity; TATSA: Triboelectric All-Textile Sensor Array; SWSs: Smart Wearable Systems.

**Table 3:** Insights regarding literature in muscular setting: authors, aim, dataset, and acquired data.

Authors	Aim	Dataset	Acquired Data
Farina et al. (2010) [47]	Proposing a novel way for interfacing myoelectric prostheses with the neuromuscular system by integrating electrodes in garments	3 HC	EMG
Samy et al. (2014) [41]	Performing sleep stage analysis with a contact-free unobtrusive system	7 HC	Respiratory rate and its variability; Leg EMG from pressure images; Sleep posture
Niijima et al. (2017) [46]	Designing and fabricating an EMG-integrated sensors cap to register EMG data of the masticatory muscles for monitoring ADL	1 HC ^1^ 3 HC ^2^	EMG (temporal muscles)
Niijima et al. (2018) [45]	Assessing the feasibility of estimating biceps fatigue using an e-textile headband	10 HC	EMG (temporal muscles)
Ozturk & Yapici (2019) [43]	Studying the performance of graphene textiles in muscular activity monitoring (acquisition of surface EMG signals from biceps brachii muscle), comparing the outcome with Ag/AgCl electrodes	1 HC	EMG (biceps brachii)
Awan et al. (2019) [44]	Presenting the fabrication of graphene-based e-textile for EMG monitoring, comparing sensing performance with commercial Ag/AgCl wet electrodes	8 HC	EMG (arm)
Yao et al. (2019) [19]	Designing and fabricating multifunctional e-textiles with mechanical and functional properties comparable with typical textiles for monitoring applications	1 HC	ECG; EMG (arm); Motion signals
Jin et al. (2019) [21]	Fabricating a metal—elastomer—nanofibers conductive material for long-term monitoring	1 HC	ECG; EMG (bicep muscle); Motion signals
Choudhry et al. (2020) [48]	Fabricating piezoresistive sensors—and studying their washability—to monitor breathing and muscular activity	1 HC	Breath pressure signal of the ribcage; Pressure signal from biceps femoris muscle
Sinha et al. (2020) [16]	Fabricating PEDOT:PSS coated electrodes to record EMG, ECG and EDA	4 HC ${}^{emg}$ 1 HC ${}^{eda}$ 1 HC ${}^{ecg}$	EDA; ECG; EMG (biceps, triceps, tibialis, and quadriceps)
Ozturk & Yapici (2020) [42]	Investigating the performance of conductive graphene textiles as surface EMG electrodes, later integrated in textile electrodes as pedometer	4 HC	sEMG

^1^ experiment 1; ^2^ experiment 2; ^*ecg*^: Electrocardiographic acquisitions; ^*eda*^: Electrodermal Activity acquisitions; ^*emg*^: Electromyographic acquisitions. **Abbreviations**. ADL: Activities of Daily Living; ECG: Electrocardiogram; EDA: Electrodermal Activity; EMG: Electromyography; HC: Healthy Controls; PEDOT:PSS: Poly(3,4-Ethyelenedioxythiophne) Polystyrene Sulfonate).

**Table 4:** Insights regarding orthopaedic literature: authors, aim, dataset, and acquired data.

Authors	Aim	Dataset	Acquired Data
Bartalesi et al. (2010) [53]	Designing, developing, and testing a wearable system to perform the real time estimation of the local curvature and the length of the spine lumbar arch	1 HC	Acceleration data; Strain signals
Lopez et al. (2010a) [24]	Describing a novel healthcare IT platform for localization and monitoring within hospital environments	5 PP	ECG; Heart rate; Angle of inclination; Activity index; Body temperature; Patient’s location; Battery level; Alert code
Lopez et al. (2010b) [25]	Presenting a medical IT platform platform based on Wireless Sensor Networks and e-textile for patients’ localization and monitoring	5 PP	ECG; Heart rate; Angle of inclination; Activity index; Body temperature; Patient’s location; Battery level; Alert code
Fevgas et al. (2010) [52]	Presenting a platform and a methodology for the rapid prototype development of e-textile applications for human activity monitoring	3 HC	Acceleration data
Della Toffola et al. (2012) [54]	Presenting a wearable system for long-term monitoring of knee kinematics in the home and community settings	1 HC	Acceleration data; Strain signals
Samy et al. (2014) [41]	Performing sleep stage analysis with a contact-free unobtrusive system	7 HC	Respiratory rate and its variability; Leg EMG from pressure images; Sleep posture
Hayashi et al. (2017) [62]	Using smart wheelchairs to monitor posture	3 HC	FS index and LL index
Li et al. (2017) [64]	Presenting an electronic dyeing method to fabricate wearable silver-based e-textile sensors for human motion monitoring and analysis	1 HC	Strain signals at heel, lower and upper knee
Vu & Kim (2018) [56]	Introducing a new approach to classify human body movements using textile sensors integrated into smart muscle pants	1 HC	Motion Signals
Lorussi et al. (2018) [60]	Developing a sensing platform constituted by wearable sensors for musculo-skeletal rehabilitation	5 HC	Knee and scapular flexion angles
Tao et al. (2018) [30]	Presenting a novel system—made up of a washable and wearable smart textile shirt, smartphone app and software desktop—for the acquisition of ECG signal, breathing rate, acceleration data for activity recognition and skin temperature	5 HC ${}^{ML}$ HC ^#^	ECG; Skin temperature; Respiratory rate; Acceleration data
Kiaghadi et al. (2018) [59]	Developing of a wearable joint sensor	1 HC	Elbow Flexion Angles; Sweat Volume
Kim et al. (2019) [37]	Developing an all-textile based pressure/strain sensor for physiological signals using 3D spacer textile	HC ^#^	Blood Pulse (wrist and neck); Finger flexion angles; Cheek motion; Pharynx motion
Park et al. (2019) [65]	Evaluation of a dynamically stretchable high-performance supercapacitor for powering an integrated sensor in an all-in-one textile system to detect various biosignals	1 HC	Strain Signals
Zhang et al. (2019) [57]	Developing a fabric E-textile for tracking active motion signals	1 HC	Motion Signals
Jang et al. (2019) [38]	Preparing a highly sensitive fiber-type strain sensor with a broad range of strain by introducing a single active layer onto the fiber	1 HC	Pulse Signals; Spinal Cord Bending Angles; Breath Signal
Ye et al. (2019) [51]	Fabricating e-textile sensors sensible to body and environmental stimuli modifying the surface of natural silks with CNTs	1 HC	Knee flexion angle; Finger flexion angle
Yao et al.(2019) [19]	Designing and fabricating multifunctional e-textiles with mechanical and functional properties comparable with typical textiles for monitoring applications	1 HC	ECG; EMG (arm); Motion signals
Jin et al. (2019) [21]	Fabricating a metal–elastomer–nanofibers conductive material for long-term monitoring	1 HC	ECG; EMG (bicep muscle); Motion signals
Raad et al. (2019) [55]	Proposing a novel Smart Glove for both live and on-demand monitoring	1 HC	Motion signals (hand and finger movement)
Amitrano et al. (2020) [61]	Presenting a novel e-textile smart sock and verifying its performances during gait analysis	3 HC	Angular velocity signals of the ankle; Foot plantar pressures
Vu & Kim (2020) [49]	Fabricating and optimizing the performance of e-textile strain sensors	1 HC	Finger flexion angles; Pharynx motion
Heo et al. (2019) [50]	Introducing, characterizing, and experimenting novel textile strain sensors based on AgNW	1 HC	Finger flexion angles
Li et al. (2020) [58]	Describing a miniature accelerometer solution integrated seamlessly within the fabric of a sleeve to monitor movement	3 HC	Elbow and knee bending angle
Tang et al. (2020) [34]	Fabricating machine-washable e-textiles with high strain sensitivity and high thermal conduction for monitoring applications	1 HC	Motion signals; Blood pulse
Garcia Patino et al. (2020) [63]	Designing a textile-based wearable platform to prevent low back pain	1 HC	Motion signals (Back movements)

^#^ number of subjects not provided; ^*ML*^: Machine Learning training set. **Abbreviations**. AgNW: Silver NanoWire; CNTs: Carbon Nanotubes; ECG: Electrocardiogram; EMG: Electromyography; FS: Forward Shift; HC: Healthy Controls; IT: Inormation Technology; LL: Lateral Lean; PP: Pathological Patients.

**Table 5:** Insights regarding literature in in respiratory field: authors, aim, dataset and acquired data.

Authors	Aim	Dataset	Acquired Data
Zieba et al. (2012) [68]	Designing a textile knitted sensor to monitor the frequency of human breathing	1 HC	Respiratory rate
Frydisiak & Zieba (2012) [69]	Designing a textile knitted sensor to monitor the frequency of human breathing	HC ^#^	Respiratory rate
Huang et al. (2013) [66]	Presenting an e-textile bedsheet to measure human respiratory rate	14 HC	Respiratory rate
Samy et al. (2014) [41]	Performing sleep stage analysis with a contact-free unobtrusive system	7 HC	Respiratory rate and its variability; Leg EMG from pressure images; Sleep posture
Liu et al. (2014) [67]	Presenting an unobtrusive on-bed respiration system	12 HC	Respiratory rate
Ramos-Garcia et al. (2016) [71]	Using a coverstitched stretch sensor in a commercial shirt to monitor respiration	3 HC	Breath signal
Ferreira et al. (2016) [15]	Presenting the design and fabrication of SWSs to prevent infants’ SIDS	HC ^#^	Body temperature; Respiratory rate; ECG
Frydisiak & Tesiorowski (2016a) [35]	Designing a smart textronic shirts for the health monitoring of elderly people	HC ^#^	Blood Pulse; Breath Signal; Skin Temperature
Frydisiak & Tesiorowski (2016b) [36]	Designing a smart textronic shirts for the health monitoring of elderly people	HC ^#^	Blood Pulse; Breath Signal; Skin Temperature
Tao et al. (2018) [30]	Presenting a novel system—made up of a washable and wearable smart textile shirt, smartphone app and software desktop—for the acquisition of ECG signal, breathing rate, acceleration data for activity recognition and skin temperature	5 HC ${}^{ML}$ HC ^#^	ECG; Skin temperature; Respiratory rate; Acceleration data
Jang et al. (2019) [38]	Preparing a highly sensitive fiber-type strain sensor with a broad range of strain by introducing a single active layer onto the fiber	1 HC	Blood Pulse; Spinal Cord Bending Angles; Breath Signal
Choundry et al. (2020) [48]	Fabricating piezoresistive sensors—and studying their washability—to monitor breathing and muscular activity	1 HC	Breath pressure signal of the ribcage; Pressure signal from biceps femoris muscle
Lian et al. (2020) [70]	Fabricating a multifunctional e-textile for multiple applications (such as diagnostics and environmental)	1 HC	Breath pressure signal
Liang et al. (2020) [32]	Developing a stable and biocompatible silk sericine carbon nanotubes (CNT) ink and demonstrating its versatile applications in flexible electronics for monitoring human biosignals	HC ^#^	ECG, Breath Signal; Hydrogen peroxide concentration
Fan et al. (2020) [39]	Developing TATSA for precise epidermal physiological signal monitoring	1 HC 1 PP	Blood Pulse & Breath Signal

^#^ number of subjects not provided; ^*ML*^: Machine Learning training set. **Abbreviations**. ECG: Electrocardiogram; EMG: Electromyography; HC: Healthy Controls; PP: Pathological Patients; SIDS: Sudden Infant Death Syndrome; SWSs: Smart Wearable Systems; TATSA: Triboelectric All-Textile Sensor Array.

**Table 6:** Insights regarding e-textile literature in other fields: authors, aim, dataset, and data.

Authors	Aim	Dataset	Acquired Data
Lopez et al. (2010a) [24]	Describing a novel healthcare IT platform for localization and monitoring within hospital environments	5 PP	ECG; Heart rate; Angle of inclination; Activity index; Body temperature; Patient’s location; Battery level; Alert code
Lopez et al. (2010b) [25]	Presenting a medical IT platform platform based on Wireless Sensor Networks and e-textile for patients’ localization and monitoring	5 PP	ECG; Heart rate; Angle of inclination; Activity index; Body temperature; Patient’s location; Battery level; Alert code
Healey et al. (2011) [76]	Presenting and validating performances of a novel e-textile sock for measuring GSR	1 HC	EDA
Liu et al. (2012) [82]	Manufacturing intelligent incontinence pants made of conductive yarns to monitor the incontinence status	HC ^#^	Volume of leaked urine
Postolache et al. (2014) [14]	Presenting a wheelchair architecture equipped with e-textiles for ECG and SKC sensing	7 HC	ECG; EDA
Mason et al. (2014) [79]	Evaluating the performance of a flexible sensor with an embedded e-textile cloth for sensing applications	1 HC	Biomedical microwave sensing
Ferreira et al. (2016) [15]	Presenting the design and fabrication of SWSs to prevent infants’ SIDS	HC ^#^	Skin temperature; Respiratory rate; ECG
Frydisiak & Tesiorowski (2016a) [35]	Designing a smart textronic shirts for the health monitoring of elderly people	HC ^#^	Blood Pulse; Breath Signal; Skin Temperature
Frydisiak & Tesiorowski (2016b) [36]	Designing a smart textronic shirts for the health monitoring of elderly people	HC ^#^	Blood Pulse; Breath Signal; Skin Temperature
Golparvar & Yapici (2017) [72]	Acquiring EOG signals with graphene textile electrodes comparing the outcome with conventional Ag/AgCl electrodes	1 HC	EOG
Golparvar & Yapici (2018a) [73]	Detecting EOG signal using textile electrodes	HC ^#^	EOG
Golparvar & Yapici (2018b) [74]	Characterization of graphene-coated electroconductive textile electrodes for EOG acquisition	4 HC 2 ME 2 HE	EOG
Lugoda et al. (2018) [77]	Fabricating temperature sensing yarns to manufacture temperature sensing garments	5 HC	Skin temperature
Chen et al. (2018) [81]	Fabricating a multifunctional e-textile for multi-detection of strain, pressure, and force maps	1 HC	Resistance signals
Haddad et al. (2018) [75]	Designing and integrating Ag/AgCl e-textile electrodes to monitor EDA comparing the outcome with standard electrodes	1 HC	EDA stimulus responses
Tao et al. (2018) [30]	Presenting a novel system—made up of a washable and wearable smart textile shirt, smartphone app and software desktop—for the acquisition of ECG signal, breathing rate, acceleration data for activity recognition and skin temperature	5 HC ${}^{ML}$ HC ^#^	ECG; Skin temperature; Respiratory rate; Acceleration data
Kiaghadi et al. (2018) [59]	Developing of a wearable joint sensor	1 HC	Elbow Flexion Angles; Sweat Volume
Kim et al. (2019) [37]	Developing an all-textile based pressure/strain sensor for physiological signals using 3D spacer textile	HC ^#^	Blood Pulse (wrist and neck); Finger flexion angles; Cheek motion; Pharynx motion
Sinha et al. (2019) [16]	Fabricating PEDOT:PSS coated electrodes to record EMG, ECG and EDA	4 HC ${}^{emg}$ 1 HC ${}^{eda}$ 1 HC ${}^{ecg}$	EDA; ECG; EMG (biceps, triceps, tibialis, and quadriceps)
Vu & Kim (2020) [49]	Fabricating and optimizing the performance of e-textile strain sensors	1 HC	Finger flexion angles; Signal of pharynx motion
Jiang et al. (2020) [78]	Integrating textile NFC antennas with temperature and humidity sensors to enable battery-free wireless sensing for monitoring purposes	1 HC	Skin Temperature; Sweat Volume
Zhao et al. (2020) [80]	Presenting a thread-based wearable nanobiosensor to detect lactate and sodium concentrations during perspiration	1 HC	Sodium and lactate concentration in human sweat
Liang et al. (2020) [32]	Developing a stable and biocompatible silk sericine carbon nanotubes (CNT) ink and demonstrating its versatile applications in flexible electronics for monitoring human biosignals	HC ^#^	ECG, Breath Signal; Hydrogen peroxide concentration

^#^ number of subjects not provided; ^*ecg*^: Electrocardiographic acquisitions; ^*eda*^: Electrodermal Activity acquisitions; ^*emg*^: Electromyographic acquisitions; ^*ML*^: Machine Learning training set. Abbreviations. ECG: Electrocardiogram; EDA: Electrodermal Activity; EMG: Electromyography; EOG: Electrooculography; HC: Healthy Controls; HE: Hypermetropic Eyes; IT: Information Technology; GSR: Galvanic Skin Response; ME: Myopic Eyes; NFC: Near Field Communication; PEDOT:PSS: Poly(3,4-Ethyelenedioxythiophne) Polystyrene Sulfonate); PP: Pathological Patients; SIDS: Sudden Infant Death Syndrome; SKC: Skin Conductivity; SWSs: Smart Wearable Systems.

## Supplementary Materials

The following are available online at https://www.mdpi.com/article/10.3390/diagnostics11122263/s1, Table S1: Insights regarding the studies included in the Review: authors, aim, material and its configuration, dataset, acquired data and clinical application.

## Abbreviations

The following abbreviations are used in this manuscript:

ADL	Activities of Daily Living
Ag/AgCl	Silver/Silver Chloride
AgNW	Silver NanoWire
AI	Artificial Intelligence
CNTs	Carbon Nanotubes
ECG	Electrocardiogram
EDA	Electrodermal Activity
EMG	Electromyography
EOG	Electrooculography
FS	Forward Shift
GSR	Galvanic Skin Response
GO	Graphene Oxide
HE	Hypermetropic Eyes
IMUs	Inertial Measurement Units
IT	Information Technology
LEVOP	Lower Extremity Venous Occlusion Plethysmography
LL	Lateral Lean
ME	Myopic Eyes
MWCNTs	Multi-Walled Carbon Nano-Tubes
NFC	Near Field Communication
PDMS	PolyDiMethylSiloxane
PEDOT:PSS	Poly(3,4-Ethyelenedioxythiophne) Polystyrene Sulfonate)
PVDF	Polyvinylidene Fluoride
SBS	Styrene–Butadiene–Styrene
SCAs	Stretchable Conductive Adhesives
SEI	Skin-Electrode Impedance
sEMG	Surface Electromyography
SIDS	Sudden Infant Death Syndrome
SKC	Skin Conductivity
SNR	Signal-to-Noise Ratio
SSCNTs	Silk-Sericine Carbon Nano-Tubes
SWCNTs	Single-Walled Carbon Nano-Tubes
SWSs	Smart Wearable Systems
TATSA	Triboelectric All-Textile Sensor Array
TPU	Termoplastic PolyUrethane
