# Supplementary material for: The E-Textile for Biomedical Applications: A Systematic Review of Literature
Source: Diagnostics (Basel). 2021 Dec 3;11(12):2263. doi: 10.3390/diagnostics11122263 (PMC8700039; doi:10.3390/diagnostics11122263)
Supplement: Supplementary file 1 [file diagnostics-11-02263-s001.zip › diagnostics-1411836-supplementary.pdf]

**Table S1.** Insights regarding the studies included in the Review: authors, aim, material and its con iguration, dataset, acquired data and clinical application.

| Authors | Aim | Material and its Configuration | Dataset | Acquired Data | Clinical Application |
|---|---|---|---|---|---|
| Bartalesi *et al.* (2010) [1] | Designing, developing, and testing a wearable system to perform the real time estimation of the local curvature and the length of the spine lumbar arch | Electrically conductive elastomer/commercial accelerometers, Lycra (rowing suit) | 1 HC | Acceleration data, strain signals | Improving the development of wearable posture capture systems |
| Lopez *et al.* (2010a) [2] | Describing a novel healthcare IT platform for localization and monitoring within hospital environments | E-textile based tape/accelerometer/thermometer, Nylon and Lycra (shirts) | 5 PP | ECG, heart rate, angle of inclination, activity index, body temperature, location, battery level, alert code | Supporting noninvasive and pervasive services demanded by future healthcare environments |
| Lopez *et al.* (2010b) [3] | Presenting a medical IT platform platform based on Wireless Sensor Networks and e-textile for patients' localization and monitoring | E-textile-based tape/accelerometer/thermometer, Nylon and Lycra (shirts) | 5 PP | ECG, heart rate, angle of inclination, activity index, body temperature, patient's location, battery level, alert code | Evaluating remote location and wearable healthcare monitoring support for hospital environments |
| Fevgas *et al.* (2010) [4] | Presenting a platform and a methodology for the rapid prototype development of e-textile applications for human activity monitoring | Commercial devices (accelerometers) | 3 HC | Acceleration data | Estimating the acceleration in different body points and to assess potential falls |
| Wu *et al.* (2010) [5] | Presenting a novel cloth electrode for ECG monitoring | Fabric with MWCNTs (electrode) | 1 HC | ECG | Performing ECG daily and long-term monitoring of the ECG signal |
| Farina *et al.* (2010) [6] | Proposing a novel way for interfacing myoelectric prostheses with the neuromuscular system by integrating electrodes in garments | Stainless steel yarns | 3 HC | EMG | Recording high-density EMG signals for myoelectric control |
| Healey *et al.* (2011) [7] | Presenting and validating performances of a novel e-textile sock for measuring GSR | Polar WearLink heart rate monitor strap (fabric electrodes), Shimmer wireless GSR sensor, soldered wires | 1 HC | EDA | Monitoring galvanic skin response from foot plant |
| Zieba *et al.* (2011) [8] | Creating new sensorical clothing structures to measure human physiological signals in a non-invasive way | Silver-woven fabric, textile laminar structure | 1 HC | ECG | Monitoring of ECG in various conditions |
| Catarino *et al.* (2012) [9] | Designing and fabricating textile integrated electrodes for ECG continuous health monitoring for disabled or elderly people | Elitex (electrode areas), polyamide/elastan (shirt).<br>The t shirt has 3 electrodes connected to the cable and the electrode areas are knitted with Elitex, which is a textured multifilament polyamide yarn with a thin silver coating | 1 HC | ECG | Monitoring ECG from a T shirt integrated with electrodes |
| Liu *et al.* (2012) [10] | Manufacturing intelligent incontinence pants made of conductive yarns to monitor the incontinence status | Nylon/spandex (elastic waistband), Nylon 66 metalized with silver (conductive yarns), Modal® and Nylon 22/20 (incontinence pants) | HC$^{\#}$ | Volume of leaked urine | Enhancing the safety and quality of life in humans by providing them with a wearable continuous detection and monitoring system |

Continued on next page

**Table S1. – continued from previous page**

| Authors | Aim | Material and its Configuration | Dataset | Acquired Data | Clinical Application |
|---|---|---|---|---|---|
| Della Toffola *et al.* (2012) [11] | Presenting a wearable system for long-term monitoring of knee kinematics in the home and community settings | Commercial perforated thermoplastic (knee attachment), conductive thread with sown sensor (e-textile), commercial textile (knee sleeve) | 1 HC | Acceleration data, strain signals | Monitoring knee flexion/extension movements during ADL; monitoring knee function during clinical trials of novel therapies |
| Zieba *et al.* (2012) [12] | Designing a textile knitted sensor to monitor the frequency of human breathing | Silver-coated polyester (yarn), cotton + elastan (shirt) | 1 HC | Respiratory Rate | Measuring the breathing rhythm frequency |
| Frydrysiak & Zieba (2012) [13] | Designing a textile knitted sensor to monitor the frequency of human breathing | Silver-coated polyester (yarn), cotton + elastan (shirt) | HC$^{\#}$ | Respiratory Rate | Monitoring the breathing rhythm of people suffering from sleep apnea |
| Huang *et al.* (2013) [14] | Presenting an e-textile bedsheet to measure human respiratory rate | Conventional fabric with conductive lines (bottom and top layer), e-textile piezoresistive fabric (middle layer) | 14 HC | Respiratory rate | Performing screenings in clinical and home environment |
| Kuroda *et al.* (2013) [15] | Prototyping an ECG sensing e-textile vest | Prototype 1: polyvinyl chloride (non-conductive yarns), stainless steel fibers (conductive yarn)<br>Prototype 2: polyester (non-conductive yarns), silver-plated nylon fibers (conductive yarn) | 1 HC | ECG | Replacing Holter ECG<br>Monitoring chronic heart patients |
| Goy *et al.* (2013) [16] | Fabricating e-textiles to monitor LEVOP | Stainless Steel (electrodes), Argenmesh, Ripstop Silver, Stainless Steel Mesh, Silver Mesh, Stretch (e-textiles) | 5 HC | LEVOP | Performing LEVOP recording |
| Samy *et al.* (2014) [17] | Performing sleep stage analysis with a contact-free unobtrusive system | Conventional fabric with conductive lines (bottom and top layer), e-textile piezoresistive fabric (middle layer) | 7 HC | Respiratory rate and its variability and leg EMG from pressure images, sleep posture | Performing screenings in clinical and home environment |
| Postolache *et al.* (2014) [18] | Presenting a wheelchair architecture equipped with e-textiles for ECG and SKC sensing | Fibers coated with conductive polymer and silver (e-textile dry electrode) | 7 HC | ECG; EDA | Collecting data for nurses/physiotherapists/physicians<br>Collecting for the wheelchair user/informal caregivers |
| Liu *et al.* (2014) [19] | Presenting an unobtrusive on-bed respiration system | Conventional fabric with conductive lines (bottom and top layer), e-textile piezoresistive fabric (middle layer) | 12 HC | Respiratory rate | Performing screenings in clinical and home environment |
| Mason *et al.* (2014) [20] | Evaluating the performance of a flexible sensor with an embedded e-textile cloth for sensing applications | Flexible fabric (cloth) supplied with conductive pathways, strain gauge, connectors, and sensor pressure points | 1 HC | Biomedical microwave sensing | Performing several sensing tasks |
| Ramos-Garcia *et al.* (2016) [21] | Using a coverstitched stretch sensor in a commercial shirt to monitor respiration | Textile-based (stretch sensor), polyester/spandex (T-shirt) | 3 HC | Breathing patterns | Capturing respiration rate in sedentary and active tasks |
| Ferreira *et al.* (2016) [22] | Presenting the design and fabrication of SWSs to prevent infants' SIDS | Silver coated textured polyamide elastic yarn (conductive yarn), electrodes, base fabric (chest belt) | HC$^{\#}$ | Skin temperature, respiratory rate, ECG | Understanding and preventing SIDS |
| Frydisiak & Tesiorowski (2016a) [23] | Designing a smart textronic shirts for the health monitoring of elderly people | Textile electrodes, not better specified | HC$^{\#}$ | Blood Pulse; Breath Signal; Skin Temperature | Monitoring heart rate, breath rate and indoor-outdoor position of elderly people |

Continued on next page

Table S1. – continued from previous page

| Authors | Aim | Material and its Configuration | Dataset | Acquired Data | Clinical Application |
|---|---|---|---|---|---|
| Frydisiak & Tesiorowski (2016b) [24] | Designing a smart textronic shirts for the health monitoring of elderly people | Textile electrodes, not better specified | HC# | Blood Pulse; Breath Signal; Skin Temperature | Monitoring heart rate, breath rate and indoor-outdoor position of elderly people |
| Li *et al.* (2017) [25] | Presenting an electronic dyeing method to fabricate wearable silver-based e-textile sensors for human motion monitoring and analysis | Silver ink (coating), polyester (e-textile) | 1 HC | Strain signals at heel, lower and upper knee | Monitoring human activity |
| Dabby *et al.* (2017) [26] | Presenting a new method for building wearable electronic and textile sensor systems directly integrated in garments to detect the heart rate | Electronic devices, stretchable copper, and waterproof elastomer (stretchable module) | 1 HC | Heart Rate | Performing heart rate long-term monitoring |
| Niijima *et al.* (2017) [27] | Designing and fabricating an EMG-integrated sensors cap to register EMG data of the masticatory muscles for monitoring ADL | PEDOT:PSS (electrodes) | 1 HC[1]<br>3 HC[2] | EMG (temporal muscles) | Monitoring the temporal activity of the masticatory muscles |
| Hayashi *et al.* (2017) [28] | Using smart wheelchairs to monitor posture | Conductive fibers (e-textile pressure sensor) | 3 HC | FS index and LL index | Notifying potentially dangerous postures |
| Golparvar & Yapici (2017) [29] | Acquiring EOG signals with graphene textile electrodes comparing the outcome with conventional Ag/AgCl electrodes | rGO (coating) | 1 HC | EOG | Monitoring EOG-related pathologies |
| Golparvar & Yapici (2018a) [30] | Detecting EOG signal using textile electrodes | rGO (coating) | HC# | EOG | Monitoring EOG-related pathologies |
| Golparvar & Yapici (2018b) [31] | Characterization of graphene-coated electroconductive textile electrodes for EOG acquisition | rGO (coating) | 4 HC<br>2 ME<br>2 HE | EOG | Monitoring EOG-related pathologies |
| Acar *et al.*(2018) [32] | Developing a single-arm ECG armband embedded with flexible graphene textiles for ECG data acquisition | GO nylon coated fibers (electrodes) | 1 HC | ECG | Recording ECG from a single arm |
| Vu & Kim (2018) [33] | Introducing a new approach to classify human body movements using textile sensors integrated into smart muscle pants | SWCNT dispersed in H2O (carbon powder-based ink), PET/SP fabric (pants) | 1 HC | Motion Signals | Monitoring human activity |
| Niijima *et al.* (2018) [34] | Assessing the feasibility of estimating biceps fatigue using an e-textile headband | PEDOT:PSS (electrodes) | 10 HC | EMG (temporal muscles) | Estimating biceps fatigue |
| Lugoda *et al.* (2018) [35] | Fabricating temperature sensing yarns to manufacture temperature sensing garments | Commercial thermistors, copper wires, textile (sensing yarns) | 5 HC | Skin temperature | Monitoring body area temperature |

Continued on next page

**Table S1. – continued from previous page**

| Authors | Aim | Material and its Configuration | Dataset | Acquired Data | Clinical Application |
|---|---|---|---|---|---|
| Lorussi *et al.* (2018) [36] | Developing a sensing platform constituted by wearable sensors for musculo-skeletal rehabilitation | Electro-conductive yarn/Lycra (knitted piezoresistive fabrics) | 5 HC | Knee and scapular flexion angles | Supporting the rehabilitation from muscoloskeletal disorders |
| Chen *et al.* (2018) [37] | Fabricating a multifunctional e-textile for multi-detection of strain, pressure, and force maps | AgNW/SBS (coating), PU (fibers) | 1 HC | Resistance signals | Detecting and monitoring several impairments/pathologies (blood pressure, RLS, neck motions, phonation) |
| Haddad *et al.* (2018) [38] | Designing and integrating Ag/AgCl e-textile electrodes to monitor EDA comparing the outcome with standard electrodes | Ag/AgCl (e-textile electrodes), cotton/nylon/polyester (fabrics) | 1 HC | EDA stimulus responses | Improving neurological disorder treatment and monitoring |
| Tao *et al.* (2018) [39] | Presenting a novel system - made up of a washable and wearable smart textile shirt, smartphone app and software desktop - for the acquisition of ECG signal, breathing rate, acceleration data for activity recognition and skin temperature | Electrodes: knitted fabric using conductive threads; Connections: conductive thread polyamide thread (Shieldtex 234/34-2 ply HCB, Statex Produktions + Vertries GmbH); Protective layer: polydimethylsiloxane and thermoplastic polyurethane | 5 $HC^{ML}$ $HC^{\#}$ | ECG; Skin temperature; Respiratory rate; Acceleration data | Continuous monitoring of ECG signal and heart rate, breath signal and rate, skin temperature and activity recognition |
| Kiaghadi *et al.* (2018) [40] | Developing of a wearable joint sensor | Sheets of cotton/lycra spandex and silver-plated nylon/elastic fiber (textile patch) | 1 HC | Elbow Flexion Angles; Sweat Volume | flexion and extension of the joints, sweat monitoring |
| Ye *et al.* (2019) [41] | Fabricating e-textile sensors sensible to body and environmental stimuli modifying the surface of natural silks with CNTs | MWCNTs/silk yarns | 1 HC | Knee flexion angle, finger flexion angles | Monitoring human activity Recognizing hand gesture |
| Li *et al.* (2019) [42] | Fabricating e-textiles depositing conducting materials thorough inkjet printing on conventional textiles for monitoring purposes | PET fibers/silver inks (e-textile) | 1 HC | ECG | Performing biometric monitoring of ECG signals |
| Ozturk & Yapici (2019) [43] | Studying the performance of graphene textiles in muscular activity monitoring (acquisition of surface EMG signals from biceps brachii muscle), comparing the outcome with Ag/AgCl electrodes | GO coated nylon cotton textile (e-textile) | 1 HC | EMG (biceps brachii) | Sensing and monitoring of muscular activity |
| Awan *et al.* (2019) [44] | Presenting the fabrication of graphene-based e-textile for EMG monitoring, comparing sensing performance with commercial Ag/AgCl wet electrodes | EOGO coated PET fabric (sensors), commercial textile (sleeve) | 8 HC | EMG (arm) | Monitoring muscular activity |

Continued on next page

**Table S1. – continued from previous page**

| Authors | Aim | Material and its Configuration | Dataset | Acquired Data | Clinical Application |
|---|---|---|---|---|---|
| Yao *et al.* (2019) [45] | Designing and fabricating multi-functional e-textiles with mechanical and functional properties comparable with typical textiles for monitoring applications | AgNW and TPU (electrodes), commercial textile (patch) | 1 HC | ECG, EMG (arm), motion signals | Assessing electrophysiological sensing, motion sensing and thermotherapy |
| Le *et al.* (2019) [46] | Comparing differences in ECG registration between silver-based textile electrodes and silver/silver-chloride gel electrodes, both integrated in a smart bra | Silver-plated nylon textile (electrodes) | 1 HC | ECG | Monitoring ECG activity |
| Jin *et al.* (2019) [47] | Fabricating a metal - elastomer - nanofibers conductive material for long-term monitoring | Fluoroelastomer + PVDF nanofibers + silver flakes (sensor electrode/wiring), polyester/spandex (sportswear) | 1 HC | ECG, EMG (bicep muscle), motion signals | Performing continuous long-term monitoring of ECG, EMG and motions in real-time |
| Raad *et al.* (2019) [48] | Proposing a novel Smart Glove for both live and on-demand monitoring | Normal stretched clothe (glove), flex and force sensors (commercial sensors), nylon (protective film) | 1 HC | Motion signals (hand and finger movement) | Monitoring patients affected by rheumatoid arthritis |
| Heo *et al.* (2019) [49] | Introducing, characterizing, and experimenting novel textile strain sensors based on AgNW | Multilayer AgNW/PDMS/PET/SP (e-textile strain sensor) | 1 HC | Finger flexion angles | Detecting hand motion<br>Discriminating grasp forces |
| Fouassier *et al.* (2019) [50] | Comparing the quality of the ECG signal registered using both a 12-lead Holter and a novel smart 12-lead ECG acquisition T-shirt | Silver yarns and hydrogel pads (electrodes), cotton fabric (T-Shirt) | 30 HC | ECG | Performing ECG long-term monitoring (Holter) |
| Kim *et al.* (2019) [51] | Developing an all-textile based pressure/strain sensor for physiological signals using 3D spacer textile | Polyurethane dispersion and carbon nanotubes ink on polyethylene terephthalate substrates (textile patch) | HC$^{\#}$ | Blood Pulse (wrist and neck); Finger flexion angles; Cheek motion; Pharynx motion | Measuring arterial pulse, motion of jugular vein and recognizing sound signal when the subject talks |
| Ko *et al.* (2019) [51] | Designing SCAs for various applications | Ag particles, MWCNTs and silicone rubber mixture | 1 HC | ECG | Monitoring of human body signals (e.g. EGC) in real-time using SCAs as electrodes |
| Park *et al.* (2019) [52] | Evaluation of a dynamically stretchable high-performance supercapacitor for powering an integrated sensor in an all-in-one textile system to detect various biosignals | MWCNT/MoO | 1 HC | Strain Signals | Detection of the strain due to joint movement and the wrist pulse |
| Zhang *et al.* (2019) [53] | Developing a fabric E-textile for tracking active motion signals | Silver and polytetrafluoroethylene yarn | 1 HC | Motion Signals | Monitoring the motion signals of the human body |
| Jang *et al.* (2019) [54] | Preparing a highly sensitive fiber-type strain sensor with a broad range of strain by introducing a single active layer onto the fiber | Coating carbon-based nanomaterials with responsive microbeads onto elastic fibers | 1 HC | Blood Pulse; Spinal Cord Bending Angles; Breath Signal | Monitoring waveforms of pulsations, respirations, and various postures of level of bending a spinal cord |

Continued on next page

**Table S1. – continued from previous page**

| Authors | Aim | Material and its Configuration | Dataset | Acquired Data | Clinical Application |
|---|---|---|---|---|---|
| Sinha *et al.* (2020) [55] | Fabricating PEDOT:PSS coated electrodes to record EMG, ECG and EDA | PEDOT:PSS (electrodes), polyester (arm sleeve) | 4 $HC^{emg}$ 1 $HC^{eda}$ 1 $HC^{ecg}$ | EDA, ECG, EMG (biceps, triceps, tibialis, and quadriceps | Performing continuous health monitoring and physiotherapy |
| Amitrano *et al.* (2020) [56] | Presenting a novel e-textile smart sock and verifying its performances during gait analysis | EeonTex (pressure sensors), commercial fabric (sock) | 3 HC | Angular velocity signals of the ankle, foot plantar pressures | Performing gait analysis and posturography |
| Choundry *et al.* (2020) [57] | Fabricating piezoresistive sensors – and studying their washability – to monitor breathing and muscular activity | Conductive sewing thread stitched on cotton fabric (layer 1), conductive aluminium (layer 2), stock paper (layer 3), cotton fabric (layer 4) | 1 HC | Breath pressure signal of the ribcage, pressure signal from biceps femoris muscle | Monitoring breathing and muscular activity<br>Detecting breathing rate |
| Lian *et al.* (2020) [58] | Fabricating a multifunctional e-textile for multiple applications (such as diagnostics and environmental) | High-density AgNW coated fabric (bottom layer), double-layered sensing fabrics with cotton mesh spacer (bottom layer) | 1 HC | Breath pressure signal | Assessing wrist heart pulse<br>Recording breath signal |
| Vu & Kim (2020) [59] | Fabricating and optimizing the performance of e-textile strain sensors | Multilayer Ag/SWCNTs/PET/SP (e-textile strain sensor) | 1 HC | Finger flexion angles, signal of pharynx motion | Monitoring – vaguely – finger or pharynx motion |
| Ozturk & Yapici (2020) [60] | Investigating the performance of conductive graphene textiles as surface EMG electrodes, later integrated in textile electrodes as pedometer | GO/nylon (conductive textile) | 4 HC | sEMG | Monitoring muscle activities during walking and pedometer |
| Jiang *et al.* (2020) [61] | Integrating textile NFC antennas with temperature and humidity sensors to enable battery-free wireless sensing for monitoring purposes | Silver yarns and cotton textile (NFC antennas), commercial sweat sensor | 1 HC | Skin Temperature; Sweat Volume | Performing personal body temperature and sweat loss monitoring from fever and sweating symptoms |
| Li *et al.* (2020) [62] | Describing a miniature accelerometer solution integrated seamlessly within the fabric of a sleeve to monitor movement | Polyimide + copper + copper wires (chip substrate), embroidered copper wires (tracking system), Lycra cuff (fastening system), polyester + cotton (fabric pockets) | 3 HC | Elbow and knee bending angle | Monitoring movement |
| Shathi *et al.* (2020a) [63] | Presenting a highly flexible and wearable e-textile for smart clothing and ECG detection | rGO/PEDOT:PSS (coating), Nylon/Lycra (fabric) | 1 HC | ECG | Detecting ECG placing the e-textile on patients' wrist |
| Shathi *et al.* (2020b) [64] | Developing e-textile electrodes for the detection of high-quality biomedical signals | rGO/PEDOT:PSS (coating), cotton (bra) | 1 HC | ECG, blood pulse | Monitoring biomedical signals using novel textile electrodes to be integrated in biomedical and health monitoring devices |
| Tang *et al.* (2020) [65] | Fabricating machine-washable e-textiles with high strain sensitivity and high thermal conduction for monitoring applications | rGO/MWCNTs/NWF (e-textile) | 1 HC | Motion signals, blood pulse | Monitoring body motion and blood pulse |
| Arquilla *et al.* (2020) [66] | Using sewn textile electrodes for ECG monitoring | Silver NPs coated nylon (thread), inextensible fabric backing (textile) | 8 HC | ECG | Integrating electrodes directly into garments for daily use |
| Garcia Patino *et al.* (2020) [67] | Designing a textile-based wearable platform to prevent low back pain | Copper wire (electronic), elastic fabric (sensors fabric) | 1 HC | Motion signals (Back movements) | Monitoring back posture to prevent or treat low back pain |

Continued on next page

**Table S1. – continued from previous page**

| Authors | Aim | Material and its Configuration | Dataset | Acquired Data | Clinical Application |
|---|---|---|---|---|---|
| Zhao et al. (2020) [68] | Presenting a thread-based wearable nanobiosensor to detect lactate and sodium concentrations during perspiration | Carbon coated cotton thread (electrode), ZnONWs (electrode coating) | 1 HC | Sodium and lactate concentration in human sweat | Quantifying patients' biochemical variables from sweat analysis |
| Liang *et al.* (2020) [69] | Developing a stable and biocompatible silk sericine carbon nanotubes (CNT) ink and demonstrating its versatile applications in flexible electronics for monitoring human biosignals | Conductive ink made of carbon nanotubes (CNTs) improved with silk sericin to allow CNT dispersion in water and improve ink biocompability | HC# | ECG, Breath Signal; Hydrogen Peroxide Concentration | Electrodes for biosignal registration (ECG, EMG, etc.); sensors for breathing rate monitoring ; electrochemical sensor for detecting hydrogene peroxide concentration |
| Fan *et al.* (2020) [70] | Developing TATSA for precise epidermal physiological signal monitoring | Stainless steel fibers (electrodes), one-ply Terylene yarns | 1 HC<br>1 PP | Blood Pulse& Breath Signal | Distinguishing between healthy individuals and those with CAD or SAS |

**Abbreviations**. # number of patients not provided; ADL: activities of daily living; AgNW: silver nanowire; CAD: Cardiovascular Disease; CNTs: carbon nanotubes; ECG: electrocardiogram; EDA: electrodermal activity; EG: ethylene glycol; EMG: electromyography;EOG: electrooculography; EOGO: edge-oxidized graphene oxide; FS: forward shift; GO: graphene oxide; GSR: Galvanic Skin Response; HC: healthy controls; IT: information technology; LEVOP: lower extremity venous occlusion plethysmography; LL: lateral lean; MWCNTs: multi-walled carbon nanotubes; NFC: near field communication ; NPs: nanoparticles; NWF: non-woven fabric; PEDOT:PSS: poly(3,4-ethyelenedioxythiophne polystyrene sulfonate); PET: polyethylene terephthalate; PP: Pathological Patients; PVDF: Polyvinylidene fluoride; rGO: reduced GO; RLS: restless legs syndrome; SAS: Sleep Apnea Syndrome; SBS: styrene-butadiene-styrene; SCAs: Stretchable Conductive Adhesives; SIDS: sudden infant death syndrome; SKC: skin conductivity; SP: spandex; SWCNTs: single-walled carbon nanotubes; SWS: smart wearable system; TATSA: Triboelectric All-Textile Sensor Array; TPU: thermoplastic polyurethane; ZnONWs : zinc-oxide nanowires.

# References


[1] R. Bartalesi, F. Lorussi, D. De Rossi, M. Tesconi, and A. Tognetti, "Wearable monitoring of lumbar spine curvature by inertial and e-textile sensory fusion," in *2010 Annual International Conference of the IEEE Engineering in Medicine and Biology*, aug 2010, pp. 6373–6376, iSSN: 1558-4615.

[2] G. Lopez, V. Custodio, and J. Ignacio Moreno, "Location-aware system for wearable physiological monitoring within hospital facilities," in *21st Annual IEEE International Symposium on Personal, Indoor and Mobile Radio Communications*, sep 2010, pp. 2609–2614, iSSN: 2166-9589.

[3] G. López, V. Custodio, and J. I. Moreno, "Lobin: e-textile and wireless-sensor-network-based platform for healthcare monitoring in future hospital environments," *IEEE Transactions on Information Technology in Biomedicine*, vol. 14, no. 6, pp. 1446–1458, nov 2010.

[4] A. Fevgas, P. Tsompanopoulou, and S. Lalis, "Rapid prototype development for studying human activity," in *XII Mediterranean Conference on Medical and Biological Engineering and Computing 2010*, ser. IFMBE Proceedings, P. D. Bamidis and N. Pallikarakis, Eds. Berlin, Heidelberg: Springer, 2010, pp. 643–646.

[5] Y.-Z. Wu, J.-X. Sun, L.-F. Li, Y.-S. Ding, and H.-A. Xu, "Performance evaluation of a novel cloth electrode," in *2010 4th International Conference on Bioinformatics and Biomedical Engineering*, jun 2010, pp. 1–5, iSSN: 2151-7622.

[6] D. Farina, T. Lorrain, F. Negro, and N. Jiang, "High-density EMG E-Textile systems for the control of active prostheses," in *2010 Annual International Conference of the IEEE Engineering in Medicine and Biology*, aug 2010, pp. 3591–3593, iSSN: 1558-4615.

[7] J. Healey, "Gsr sock: a new e-textile sensor prototype," in *2011 15th Annual International Symposium on Wearable Computers*, jun 2011, pp. 113–114, iSSN: 2376-8541.

[8] J. Zięba, M. Frydrysiak, L. Tesiorowski, and M. Tokarska, "Textronic clothing to ECG measurement," in *2011 IEEE International Symposium on Medical Measurements and Applications*, may 2011, pp. 559–563.

[9] A. Catarino, H. Carvalho, M. J. Dias, T. Pereira, O. Postolache, and P. S. Girão, "Continuous health monitoring using E-textile integrated biosensors," in *2012 International Conference and Exposition on Electrical and Power Engineering*, oct 2012, pp. 605–609.

[10] R. Liu, S. Wang, and T. T. Lao, "A novel solution of monitoring incontinence status by conductive yarn and advanced seamless knitting techniques," *Journal of Engineered Fibers and Fabrics*, vol. 7, no. 4, p. 155892501200700415, dec 2012. [Online]. Available: https://doi.org/10.1177/155892501200700415

[11] L. D. Toffola, S. Patel, M. Y. Ozsecen, R. Ramachandran, and P. Bonato, "A wearable system for long-term monitoring of knee kinematics," in *Proceedings of 2012 IEEE-EMBS International Conference on Biomedical and Health Informatics*, jan 2012, pp. 188–191, iSSN: 2168-2208.

[12] J. Zięba, M. Frydrysiak, and J. Błaszczyk, "Textronic clothing with resistance textile sensor to monitoring frequency of human breathing," in *2012 IEEE International Symposium on Medical Measurements and Applications Proceedings*, may 2012, pp. 1–6.

[13] M. Frydrysiak and J. Zięba, "Textronic sensor for monitoring respiratory rhythm," *Fibres and Textiles in Eastern Europe*, vol. 91, no. 2, pp. 74–78, 2012.

[14] M.-C. Huang, W. Xu, J. Liu, L. Samy, A. Vajid, N. Alshurafa, and M. Sarrafzadeh, "Inconspicuous on-bed respiratory rate monitoring," in *Proceedings of the 6th International Conference on PErvasive Technologies Related to Assistive Environments*, ser. PETRA '13. Rhodes, Greece: Association for Computing Machinery, may 2013, pp. 1–8. [Online]. Available: https://doi.org/10.1145/2504335.2504353

[15] T. Kuroda, K. Hirano, K. Sugimura, S. Adachi, H. Igarashi, K. Ueshima, H. Nakamura, M. Nambu, and T. Doi, "Applying NISHIJIN historical textile technique for e-Textile," in *2013 35th Annual International Conference of the IEEE Engineering in Medicine and Biology Society (EMBC)*, jul 2013, pp. 1226–1229, iSSN: 1558-4615.

[16] C. B. Goy, J. M. Dominguez, M. A. Gómez López, R. E. Madrid, and M. C. Herrera, "Electrical characterization of conductive textile materials and its evaluation as electrodes for venous occlusion plethysmography," *Journal of Medical Engineering & Technology*, vol. 37, no. 6, pp. 359–367, aug 2013. [Online]. Available: https://doi.org/10.3109/03091902.2013.812689

[17] L. Samy, M.-C. Huang, J. J. Liu, W. Xu, and M. Sarrafzadeh, "Unobtrusive sleep stage identification using a pressure-sensitive bed sheet," *IEEE Sensors Journal*, vol. 14, no. 7, pp. 2092–2101, jul 2014.

[18] O. Postolache, V. Viegas, J. M. Dias Pereira, D. Vinhas, P. S. Girão, and G. Postolache, "Toward developing a smart wheelchair for user physiological stress and physical activity monitoring," in *2014 IEEE International Symposium on Medical Measurements and Applications (MeMeA)*, jun 2014, pp. 1–6.

[19] J. J. Liu, M.-C. Huang, W. Xu, X. Zhang, L. Stevens, N. Alshurafa, and M. Sarrafzadeh, "Breathsens: a continuous on-bed respiratory monitoring system with torso localization using an unobtrusive pressure sensing array," *IEEE Journal of Biomedical and Health Informatics*, vol. 19, no. 5, pp. 1682–1688, sep 2015.

[20] A. Mason, S. Wylie, O. Korostynska, L. E. Cordova-Lopez, and A. I. Al-Shamma'a, "Flexible e-textile sensors for real-time health monitoring at microwave frequencies," *International Journal on Smart Sensing and Intelligent Systems*, vol. 7, no. 1, dec 2017. [Online]. Available: https://doi.org/10.21307/ijssis-2017-644

[21] R. I. Ramos-Garcia, F. Da Silva, Y. Kondi, E. Sazonov, and L. E. Dunne, "Analysis of a coverstitched stretch sensor for monitoring of breathing," in *2016 10th International Conference on Sensing Technology (ICST)*, nov 2016, pp. 1–6, iSSN: 2156-8073.

[22] A. G. Ferreira, D. Fernandes, S. Branco, J. L. Monteiro, J. Cabral, A. P. Catarino, and A. M. Rocha, "A smart wearable system for sudden infant death syndrome monitoring," in *2016 IEEE International Conference on Industrial Technology (ICIT)*, mar 2016, pp. 1920–1925.

[23] M. Frydrysiak and L. Tesiorowski, "Wearable textronic system for protecting elderly people," in *2016 IEEE International Symposium on Medical Measurements and Applications (MeMeA)*. Benevento, Italy: IEEE, may 2016, pp. 1–6. [Online]. Available: http://ieeexplore.ieee.org/document/7533699/

[24] ——, "Health monitoring system for protecting elderly people," in *2016 International Multidisciplinary Conference on Computer and Energy Science (SpliTech)*. Split, Croatia: IEEE, jul 2016, pp. 1–6. [Online]. Available: http://ieeexplore.ieee.org/document/7555935/

[25] Y. Li, Y. Li, M. Su, W. Li, Y. Li, H. Li, X. Qian, X. Zhang, F. Li, and Y. Song, "Electronic textile by dyeing method for multiresolution physical kineses monitoring," *Advanced Electronic Materials*, vol. 3, no. 10, p. 1700253, 2017. [Online]. Available: https://onlinelibrary.wiley.com/doi/abs/10.1002/aelm.201700253

[26] N. Dabby, A. Aleksov, E. Lewallen, S. Oster, R. Fygenson, B. Lathrop, M. Bynum, M. Samady, S. Klein, and S. Girouard, "A scalable process for manufacturing integrated, washable smart garments applied to heart rate monitoring," in *Proceedings of the 2017 ACM International Symposium on Wearable Computers*, ser. ISWC '17. Maui, Hawaii: Association for Computing Machinery, sep 2017, pp. 38–41. [Online]. Available: https://doi.org/10.1145/3123021.3123045

[27] A. Niijima, T. Isezaki, R. Aoki, T. Watanabe, and T. Yamada, "Hitoecap: wearable emg sensor for monitoring masticatory muscles with pedot-pss textile electrodes," in *Proceedings of the 2017 ACM International Symposium on Wearable Computers*, ser. ISWC '17. Maui, Hawaii: Association for Computing Machinery, sep 2017, pp. 215–220. [Online]. Available: https://doi.org/10.1145/3123021.3123068

[28] C. Hayashi, Y. Enokibori, and K. Mase, "Harmless line-oriented sensing point reduction for non-categorical sitting posture score," in *Proceedings of the 2017 ACM International Joint Conference on Pervasive and Ubiquitous Computing and Proceedings of the 2017 ACM International Symposium on Wearable Computers*, ser. UbiComp '17. Maui, Hawaii: Association for Computing Machinery, sep 2017, pp. 61–64. [Online]. Available: https://doi.org/10.1145/3123024.3123083

[29] A. J. Golparvar and M. Kaya Yapici, "Wearable graphene textile-enabled EOG sensing," in *2017 IEEE SENSORS*, oct 2017, pp. 1–3.

[30] A. J. Golparvar and M. K. Yapici, "Graphene-coated wearable textiles for EOG-based human-computer interaction," in *2018 IEEE 15th International Conference on Wearable and Implantable Body Sensor Networks (BSN)*, mar 2018, pp. 189–192, iSSN: 2376-8894.

[31] ——, "Electrooculography by wearable graphene textiles," *IEEE Sensors Journal*, vol. 18, no. 21, pp. 8971–8978, nov 2018.

[32] G. Acar, O. Ozturk, and M. K. Yapici, "Wearable graphene nanotextile embedded smart armband for cardiac monitoring," in *2018 IEEE SENSORS*. New Delhi: IEEE, oct 2018, pp. 1–4. [Online]. Available: https://ieeexplore.ieee.org/document/8589800/

[33] C. Vu and J. Kim, "Human motion recognition using e-textile sensor and adaptive neuro-fuzzy inference system," *Fibers and Polymers*, vol. 19, no. 12, pp. 2657–2666, dec 2018. [Online]. Available: http://link.springer.com/10.1007/s12221-018-8019-0

[34] A. Niijima, T. Isezaki, R. Aoki, T. Watanabe, and T. Yamada, "Biceps fatigue estimation with an E-textile headband," in *Proceedings of the 2018 ACM International Symposium on Wearable Computers*, ser. ISWC '18. Singapore, Singapore: Association for Computing Machinery, oct 2018, pp. 222–223. [Online]. Available: https://doi.org/10.1145/3267242.3267281

[35] P. Lugoda, T. Hughes-Riley, C. Oliveira, R. Morris, and T. Dias, "Developing novel temperature sensing garments for health monitoring applications," *Fibers*, vol. 6, no. 3, p. 46, sep 2018. [Online]. Available: https://www.mdpi.com/2079-6439/6/3/46

[36] F. Lorussi, I. Lucchese, A. Tognetti, and N. Carbonaro, "A wearable system for remote monitoring of the treatments of musculoskeletal disorder," in *2018 IEEE International Conference on Smart Computing (SMARTCOMP)*, jun 2018, pp. 362–367.

[37] S. Chen, S. Liu, P. Wang, H. Liu, and L. Liu, "Highly stretchable fiber-shaped e-textiles for strain/pressure sensing, full-range human motions detection, health monitoring, and 2D force mapping," *Journal of Materials Science*, vol. 53, no. 4, pp. 2995–3005, feb 2018. [Online]. Available: https://doi.org/10.1007/s10853-017-1644-y

[38] P. A. Haddad, A. Servati, S. Soltanian, F. Ko, and P. Servati, "Breathable dry silver/silver chloride electronic textile electrodes for electrodermal activity monitoring," *Biosensors*, vol. 8, no. 3, p. 79, sep 2018. [Online]. Available: https://www.mdpi.com/2079-6374/8/3/79

[39] X. Tao, T.-H. Huang, C.-L. Shen, Y.-C. Ko, G.-T. Jou, and V. Koncar, "Bluetooth low energy-based washable wearable activity motion and electrocardiogram textronic monitoring and communicating system," *Advanced Materials Technologies*, vol. 3, no. 10, p. 1700309, oct 2018. [Online]. Available: https://onlinelibrary.wiley.com/doi/10.1002/admt.201700309

[40] A. Kiaghadi, M. Baima, J. Gummeson, T. Andrew, and D. Ganesan, "Fabric as a sensor: towards unobtrusive sensing of human behavior with triboelectric textiles," in *Proceedings of the 16th ACM Conference on Embedded Networked Sensor Systems*. Shenzhen China: ACM, nov 2018, pp. 199–210. [Online]. Available: https://dl.acm.org/doi/10.1145/3274783.3274845

[41] C. Ye, J. Ren, Y. Wang, W. Zhang, C. Qian, J. Han, C. Zhang, K. Jin, M. J. Buehler, D. L. Kaplan, and S. Ling, "Design and fabrication of silk templated electronic yarns and applications in multifunctional textiles," *Matter*, vol. 1, no. 5, pp. 1411–1425, nov 2019. [Online]. Available: https://www.sciencedirect.com/science/article/pii/S2590238519301201

[42] B. M. Li, I. Kim, Y. Zhou, A. C. Mills, T. J. Flewwellin, and J. S. Jur, "Kirigami-inspired textile electronics: k. I. T. E." *Advanced Materials Technologies*, vol. 4, no. 11, p. 1900511, 2019. [Online]. Available: https://onlinelibrary.wiley.com/doi/abs/10.1002/admt.201900511

[43] O. Ozturk and M. K. Yapici, "Muscular activity monitoring and surface electromyography (Semg) with graphene textiles," in *2019 IEEE SENSORS*, oct 2019, pp. 1–4, iSSN: 2168-9229.

[44] F. Awan, Y. He, L. Le, L.-L. Tran, H.-D. Han, and L. P. Nguyen, "Electromyography acquisition system using graphene-based e-textiles," in *2019 International Symposium on Electrical and Electronics Engineering (ISEE)*, oct 2019, pp. 59–62.

[45] S. Yao, J. Yang, F. R. Poblete, X. Hu, and Y. Zhu, "Multifunctional electronic textiles using silver nanowire composites," *ACS Applied Materials & Interfaces*, vol. 11, no. 34, pp. 31 028–31 037, aug 2019. [Online]. Available: https://pubs.acs.org/doi/10.1021/acsami.9b07520

[46] K. Le, A. Servati, F. Ko, and P. Servati, "Signal quality analysis of electrocardiogram textile electrodes for smart apparel applications," in *2019 IEEE International Flexible Electronics Technology Conference (IFETC)*, aug 2019, pp. 1–3.

[47] H. Jin, M. O. G. Nayeem, S. Lee, N. Matsuhisa, D. Inoue, T. Yokota, D. Hashizume, and T. Someya, "Highly durable nanofiber-reinforced elastic conductors for skin-tight electronic textiles," *ACS Nano*, vol. 13, no. 7, pp. 7905–7912, jul 2019. [Online]. Available: https://doi.org/10.1021/acsnano.9b02297

[48] M. Raad, M. Deriche, A. Hafeedh, H. Almasawa, K. Jofan, H. Alsakkaf, A. Bahumran, and M. Salem, "An iot based wearable smart glove for remote monitoring of rheumatoid arthritis patients:," in *Proceedings of the 12th International Joint Conference on Biomedical Engineering Systems and Technologies.* Prague, Czech Republic: SCITEPRESS - Science and Technology Publications, 2019, pp. 224–228. [Online]. Available: http://www.scitepress.org/DigitalLibrary/Link.aspx?doi=10.5220/0007573302240228

[49] J. S. Heo, H. H. Shishavan, R. Soleymanpour, J. Kim, and I. Kim, "Textile-based stretchable and flexible glove sensor for monitoring upper extremity prosthesis functions," *IEEE Sensors Journal*, vol. 20, no. 4, pp. 1754–1760, feb 2020.

[50] D. Fouassier, X. Roy, A. Blanchard, and J.-S. Hulot, "Assessment of signal quality measured with a smart 12-lead ECG acquisition T-shirt," *Annals of Noninvasive Electrocardiology*, vol. 25, no. 1, p. e12682, 2020. [Online]. Available: https://onlinelibrary.wiley.com/doi/abs/10.1111/anec.12682

[51] K. Kim, M. Jung, S. Jeon, and J. Bae, "Robust and scalable three-dimensional spacer textile pressure sensor for human motion detection," *Smart Materials and Structures*, vol. 28, no. 6, p. 065019, jun 2019. [Online]. Available: https://iopscience.iop.org/article/10.1088/1361-665X/ab1adf

[52] H. Park, J. W. Kim, S. Y. Hong, G. Lee, H. Lee, C. Song, K. Keum, Y. R. Jeong, S. W. Jin, D. S. Kim, and J. S. Ha, "Dynamically stretchable supercapacitor for powering an integrated biosensor in an all-in-one textile system," *ACS Nano*, vol. 13, no. 9, pp. 10 469–10 480, sep 2019. [Online]. Available: https://pubs.acs.org/doi/10.1021/acsnano.9b04340

[53] X. Zhang, J. Wang, Y. Xing, and C. Li, "Woven wearable electronic textiles as self-powered intelligent tribo-sensors for activity monitoring," *Global Challenges*, vol. 3, no. 12, p. 1900070, dec 2019. [Online]. Available: https://onlinelibrary.wiley.com/doi/10.1002/gch2.201900070

[54] S. Jang, J. Kim, D. W. Kim, J. W. Kim, S. Chun, H. J. Lee, G.-R. Yi, and C. Pang, "Carbon-based, ultraelastic, hierarchically coated fiber strain sensors with crack-controllable beads," *ACS Applied Materials & Interfaces*, vol. 11, no. 16, pp. 15 079–15 087, apr 2019. [Online]. Available: https://pubs.acs.org/doi/10.1021/acsami.9b03204

[55] S. K. Sinha, H. F. Posada-Quintero, Y. Noh, C. Allen, R. Daniels, K. H. Chon, L. Sloan, and G. A. Sotzing, "Integrated dry poly(3,4-ethylenedioxythiophene):polystyrene sulfonate electrodes on finished textiles for continuous and simultaneous monitoring of electrocardiogram, electromyogram and electrodermal activity," *Flexible and Printed Electronics*, vol. 5, no. 3, p. 035009, sep 2020. [Online]. Available: https://iopscience.iop.org/article/10.1088/2058-8585/abad89

[56] F. Amitrano, A. Coccia, C. Ricciardi, L. Donisi, G. Cesarelli, E. M. Capodaglio, and G. D'Addio, "Design and validation of an e-textile-based wearable sock for remote gait and postural assessment," *Sensors*, vol. 20, no. 22, p. 6691, jan 2020. [Online]. Available: https://www.mdpi.com/1424-8220/20/22/6691

[57] N. A. Choudhry, A. Rasheed, S. Ahmad, L. Arnold, and L. Wang, "Design, development and characterization of textile stitch-based piezoresistive sensors for wearable monitoring," *IEEE Sensors Journal*, vol. 20, no. 18, pp. 10 485–10 494, sep 2020.

[58] Y. Lian, H. Yu, M. Wang, X. Yang, Z. Li, F. Yang, Y. Wang, H. Tai, Y. Liao, J. Wu, X. Wang, Y. Jiang, and G. Tao, "A multifunctional wearable E-textile via integrated nanowire-coated fabrics," *Journal of Materials Chemistry C*, vol. 8, no. 25, pp. 8399–8409, jul 2020. [Online]. Available: https://pubs.rsc.org/en/content/articlelanding/2020/tc/d0tc00372g

[59] C. C. Vu and J. Kim, "Highly sensitive e-textile strain sensors enhanced by geometrical treatment for human monitoring," *Sensors*, vol. 20, no. 8, p. 2383, jan 2020. [Online]. Available: https://www.mdpi.com/1424-8220/20/8/2383

[60] O. Ozturk and M. K. Yapici, "Surface electromyography with wearable graphene textiles," *IEEE Sensors Journal*, vol. 21, no. 13, pp. 14 397–14 406, jul 2021.

[61] Y. Jiang, K. Pan, T. Leng, and Z. Hu, "Smart textile integrated wireless powered near field communication body temperature and sweat sensing system," *IEEE Journal of Electromagnetics, RF and Microwaves in Medicine and Biology*, vol. 4, no. 3, pp. 164–170, sep 2020.

[62] M. Li, R. Torah, H. Nunes-Matos, Y. Wei, S. Beeby, J. Tudor, and K. Yang, "Integration and testing of a three-axis accelerometer in a woven e-textile sleeve for wearable movement monitoring," *Sensors*, vol. 20, no. 18, p. 5033, jan 2020. [Online]. Available: https://www.mdpi.com/1424-8220/20/18/5033

[63] M. Akter Shathi, C. Minzhi, N. A. Khoso, H. Deb, A. Ahmed, and W. Sai Sai, "All organic graphene oxide and Poly (3, 4-ethylene dioxythiophene) - Poly (Styrene sulfonate) coated knitted textile fabrics for wearable electrocardiography (Ecg) monitoring," *Synthetic Metals*, vol. 263, p. 116329, may 2020. [Online]. Available: https://www.sciencedirect.com/science/article/pii/S0379677919309804

[64] M. A. Shathi, M. Chen, N. A. Khoso, M. T. Rahman, and B. Bhattacharjee, "Graphene coated textile based highly flexible and washable sports bra for human health monitoring," *Materials & Design*, vol. 193, p. 108792, aug 2020. [Online]. Available: https://www.sciencedirect.com/science/article/pii/S0264127520303269

[65] Z. Tang, D. Yao, D. Du, and J. Ouyang, "Highly machine-washable e-textiles with high strain sensitivity and high thermal conduction," *Journal of Materials Chemistry C*, vol. 8, no. 8, pp. 2741–2748, feb 2020. [Online]. Available: https://pubs.rsc.org/en/content/articlelanding/2020/tc/c9tc06155j

[66] K. Arquilla, A. K. Webb, and A. P. Anderson, "Textile electrocardiogram (Ecg) electrodes for wearable health monitoring," *Sensors*, vol. 20, no. 4, p. 1013, jan 2020. [Online]. Available: https://www.mdpi.com/1424-8220/20/4/1013

[67] A. García Patiño, M. Khoshnam, and C. Menon, "Wearable device to monitor back movements using an inductive textile sensor," *Sensors*, vol. 20, no. 3, p. 905, jan 2020. [Online]. Available: https://www.mdpi.com/1424-8220/20/3/905

[68] C. Zhao, X. Li, Q. Wu, and X. Liu, "A thread-based wearable sweat nanobiosensor," *Biosensors and Bioelectronics*, vol. 188, p. 113270, sep 2021. [Online]. Available: https://www.sciencedirect.com/science/article/pii/S0956566321003079

[69] X. Liang, H. Li, J. Dou, Q. Wang, W. He, C. Wang, D. Li, J. Lin, and Y. Zhang, "Stable and biocompatible carbon nanotube ink mediated by silk protein for printed electronics," *Advanced Materials*, vol. 32, no. 31, p. 2000165, aug 2020. [Online]. Available: https://onlinelibrary.wiley.com/doi/10.1002/adma.202000165

[70] W. Fan, Q. He, K. Meng, X. Tan, Z. Zhou, G. Zhang, J. Yang, and Z. L. Wang, "Machine-knitted washable sensor array textile for precise epidermal physiological signal monitoring," *Science Advances*, vol. 6, no. 11, p. eaay2840, mar 2020. [Online]. Available: https://www.science.org/doi/10.1126/sciadv.aay2840
